# Direct AKT activation in tumor-infiltrating lymphocytes markedly increases interferon-γ (IFN-γ) for the regression of tumors resistant to PD-1 checkpoint blockade

**DOI:** 10.1038/s41598-022-23016-z

**Published:** 2022-11-02

**Authors:** François Santinon, Bennani Fatima Ezzahra, Meriem Bachais, Alain Sarabia Pacis, Christopher E. Rudd

**Affiliations:** 1 Département de Médecine, Université de Montréal, Montréal, QC H3C 3J7 Canada; 2 Division of Immunology-Oncology, Centre de Recherche Hopital Maisonneuve-Rosemont, Montréal, QC H1T 2M4 Canada; 3 Canadian Centre for Computational Genomics, McGill University, Montréal, QC H3A 0G1 Canada

**Keywords:** Cancer, Immunology

## Abstract

PD-1 immune checkpoint blockade against inhibitory receptors such as receptor programmed cell death-1 (PD-1), has revolutionized cancer treatment. Effective immune reactivity against tumour antigens requires the infiltration and activation of tumour-infiltrating T-cells (TILs). In this context, ligation of the antigen-receptor complex (TCR) in combination with the co-receptor CD28 activates the intracellular mediator AKT (or PKB, protein kinase B) and its downstream targets. PD-1 inhibits the activation of AKT/PKB. Given this, we assessed whether the direct activation of AKT might be effective in activating the immune system to limit the growth of tumors that are resistant to PD-1 checkpoint blockade. We found that the small molecule activator of AKT (SC79) limited growth of a B16 tumor and an EMT-6 syngeneic breast tumor model that are poorly responsive to PD-1 immunotherapy. In the case of B16 tumors, direct AKT activation induced (i) a reduction of suppressor regulatory (Treg) TILs and (ii) an increase in effector CD8+ TILs. SC79 in vivo therapy caused a major increase in the numbers of CD4+ and CD8+ TILs to express interferon-γ (IFN-γ). This effect on IFN-γ expression distinguished responsive from non-responsive anti-tumor responses and could be recapitulated ex vivo with human T-cells. In CD4+FoxP3+Treg TILs, AKT induced IFN-γ expression was accompanied by a loss of suppressor activity, the conversation to CD4^+^ helper Th1-like TILs and a marked reduction in phospho-SHP2. In CD8+ TILs, we observed an increase in the phospho-activation of PLC-γ. Further, the genetic deletion of the transcription factor T-bet (*Tbx21*) blocked the increased IFN-γ expression on all subsets while ablating the therapeutic benefits of SC79 on tumor growth. Our study shows that AKT activation therapy acts to induce IFN-γ on CD4 and CD8 TILs that is accompanied by the intra-tumoral conversation of suppressive Tregs into CD4^+^Th1-like T-cells and augmented CD8 responses.

## Introduction

The advent of immunotherapy has ushered in a new era for cancer treatment. Immune checkpoint blockade (ICB) employs monoclonal antibodies (mAbs) that obstruct the binding of inhibitory receptors (IRs) on T cells to their natural ligands, often expressed by cancer cells^1–3^. Blockade of cytotoxic T-lymphocyte–associated antigen 4 (*CTLA*-*4*) and programmed death 1 (PD-1) or the PD-1 ligand, PD-L1 have achieved survival rates of 30–50% in various cancers such as non-small cell lung carcinoma (NSCLC), melanoma, and bladder cancer^4,5^. However, most patients are not cured underscoring the need for alternate or complementary clinical interventions.

In this context, successful ICB has been characterised by an increase in the presence of CD8+ cytolytic effector T-cells and/or the reduced presence of suppressor cells such as regulatory T-cells (Tregs)^6^. High tumor infiltration by Tregs and a low ratio of effector T cells (Teffs) to Tregs is associated with a poor outcome in solid tumors^7^, while a high Teff/Treg cell ratio is associated with robust responses to immunotherapy^8^. To date, anti-CD25 or anti-CTLA-4 antibodies have been used in vivo to eliminate Tregs via FcγRIII+ phagocytic cells^9–12^. Others have used anti-CD25 immunotoxins to deplete this subset^13^. However, success has been variable since the efficacy depends on the expression of surface receptors and immune depletion. Although anti-CTLA-4 depletes Tregs in mouse studies^14^, anti-CTLA-4 immunotherapy does not deplete FoxP3+ Tregs in human cancers^15^.

To this end, it should be of benefit to devise an alternate method of Treg depletion. In this context, successful anti-PD-1 therapy depends on the expression of the co-receptor CD28 and its ability to generate intracellular signals^16^. We and others previously showed the antigen receptor (TCR) generates signals via protein-tyrosine kinases^17–19^, while CD28 binds to the adaptor protein GRb2^20^ and the lipid kinase, phosphatidyl inositol kinase (PI-3K) which can activate the serine/threonine kinase AKT/protein kinase B (PKB)^21,22^. Conversely, PD-1 inhibits the activation of the AKT and Ras pathways in T-cells^23^. There are three isoforms of AKT, each possessing a pleckstrin homology domain that binds either PIP_3_ PtdIns(3,4,5-*P*_3_) or PIP_2_ PtdIns(3,4-*P*_2_)^24^. PI 3K generates PIP_3_ from PIP_2_. AKT is phosphorylated by the activating kinases, phosphoinositide-dependent kinase 1 (PDPK1) on threonine 308 and the mammalian target of rapamycin complex 2 (mTOR2) at serine 473^25^. AKT then acts downstream to affect multiple pathways^26,27^. In this context, phospho-AKT has been considered as a therapeutic target for the treatment of malignant tumors where phosphorylation of AKT at Ser473 has been reported to promote breast cancer metastasis^28^. Upon PI-3K activation, AKT phosphorylates FOXO1, causing its exportation from the nucleus into the cytoplasm^29^. FOXO1 binds to the consensus sequence in the *Foxp3* promoter and directly activates its expression^30^. Previous studies had implicated AKT in the survival of T cells during the effector-to-memory cell transition^31^, where inhibitors of AKT have been used therapeutically to conserve memory T-cells in adoptive cell therapy (ACT)^32^.

Given this background, we hypothesized that the direct activation of AKT with small molecule (SM) activators might bypass the need for biologics and overcome resistance to ICB. To this end, a small molecule activator of AKT, SC79 has been reported, but to our knowledge, not tested in detail in tumor models^33^. In this study, we show for the first time that SM AKT activation acts to limit the growth of B16 tumors resistant to anti-PD-1 therapy broadly causing a profound increase in the expression of the effector cytokine interferon-γ (IFN-γ) on CD4 and CD8 TILs. In the case of Tregs, the expression of IFN-γ lead to the loss of Tregs and the intra-tumoral conversation of Tregs to CD4^+^Th1-like TILs. Our findings indicate that AKT activation by a small molecule is a novel potential approach for depleting Tregs and activating CD8+IFN-γ TILs in the destruction of tumor cells.

## Results

### Small molecules activation of AKT regresses anti-PD-1 resistant B16 tumors

**Figure 1:**
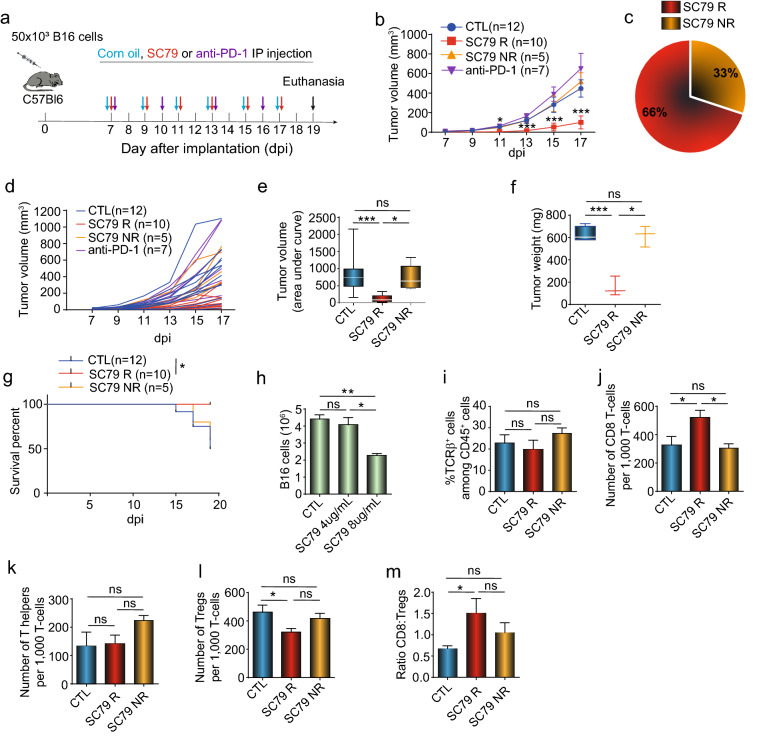
SC79 decreases anti-PD-1 resistant B16 tumor with an increase of the tumor infiltrated CD8-T-cell: Treg ratio: (**a**) Regime of treatment. (**b**) SC79 reduces B16 tumor growth. Control group (CTL,12), SC79 responder (SC79 R; 10), SC79 no responder (SC79 NR,5) and anti-PD-1 treated mice (anti-PD-1; 7). Data were pooled from 3 independent experiments (n = 3). (**c**) Proportion of the responder and non-responder mice to the SC79 treatment (n = 3). (**d**) Spider plots of SC79 reduction of B16 tumor growth (n = 3). (**e**) Histogram showing the tumor value using a measure (statistical area under the curve of the tumor growth for each group). (**f**) Tumor weight was measured on day 19 after implantation. Data show the representative result of three independent experiments (n = 3). (**g**) Survival curve. Mice were considered dead if dead or if the tumor size exceeded 1500 mm^3^ (which then required culling of mice) (n = 3). (**h**) Survival of B16 cells in in vitro culture with SC79 (n = 3). (**i**) Frequency of TCRβ+ T-cells within the CD45+ TIL population. (**j**) Number of CD8+ TILs is increased in responder but not in non-responder tumors (n = 5). (**k**) Number of CD4+ FoxP3− TILs in control, responder and non-responder tumors (n = 5). (**I**) Number of CD4+FoxP3+ Treg TILs is decreased in responder but not in non-responder tumors (n = 5). (**m**) CD8 T-cell to Treg ratio in TILs. CD8+ TILs increased in responder but not in non-responder tumors (n = 5). Results from panels i to m were obtained from 5 control tumors, 4 SC79 responder tumors and 3 SC79 non-responder tumors. For statistical analyses, parametric unpaired t-test on the area under the curve from each mouse, Mantel-cox test and one-way ANOVA were used. *p < 0.05, ***p < 0.001.

The small molecule activator SC79 potentiates T-cell responses but has not been tested in tumor models^33^. C57Bl/6 mice were injected subcutaneously with a B16 melanoma variant for 7 days followed by treatment with corn oil (control), SC79 (50ug/mouse) or anti-PD-1 until day 19 (Fig. 1a). The B16 melanoma implants increased in size and were seen to be resistant to anti-PD-1 (J43) therapy. By contrast, injections of SC79 significantly reduced B16 tumor growth in 10/15 mice (i.e., responsive) as defined by a reduction in tumor size by more than 50 per cent relative to untreated controls (Fig. 1b). We observed that 66 per cent of mice treated with SC79 showed a reduction in tumor weight (Fig. 1b,c). Spider graphs further underscored this observation by showing the reduction in tumor growth for individual mice (Fig. 1d). The decrease was seen in terms of tumor volume (Fig. 1e) and tumor weight (Fig. 1f) and resulted in an increase in mouse survival (Fig. 1g). A significant reduction in tumor size was also seen when tumors of responsive and non-responsive mice were grouped together in each treatment group (Fig. S1a). Further, SC79 reduced tumor volume in the EMT-6 syngeneic triple-negative breast cancer tumor model (Fig. S1b). In the case of B16 cells, the in vitro incubation of B16 cells with 4ug/ml or less of SC79 did not affect the presence or viability of cells (Fig. 1h). This concentration is higher than would be present in mice at a dose of 50ug/mouse. Further, in the B16 model, there was no obvious increase in the percentage of TCRβ+ T-cells relative to the CD45+ T-cells in mice (Fig. 1i). However, there was a statistically significant increase in the presence of CD8+ TILs within the TCRβ+ TIL subset in SC79 responder mice relative to control and non-responder mice (Fig. 1j). Further, although there was no change in the presence of CD4+ FoxP3− helper TILs (Fig. 1k), there was a statistically significant decrease in CD4+FoxP3+ Tregs in the SC79 responder mice relative to control and non-responder mice (Fig. 1I), This difference was also reflected by an increase in the ratio of CD8+ T-cells to Tregs in SC79R mice (Fig. 1m). These data showed that SC79 small molecule activation of AKT regress tumors that are otherwise poorly responsive to anti-PD1 therapy and further, this process is characterised by an increase in CD8+ TILs and a reduction in the presence of CD4+FoxP3+ Treg TILs.

**Figure 2:**
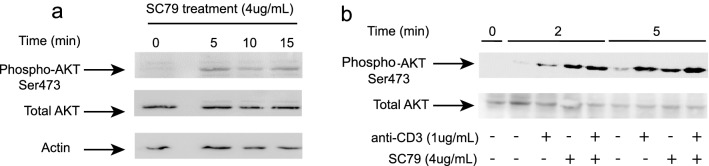
SC79 induces AKT serine 473 phosphorylation in T-cells. Immunoblot of mouse T-cells, either resting, anti-CD3 or SC79 or combination treatment with the anti-pAKT and anti-AKT (n = 3). (**a**) Time course of SC79 activation of AKT in T-cells (time 015 min). (**b**) SC79 induced AKT p473 is similar to that induced by anti-CD3 over 10 min.

We also assessed whether SC79 could increase the activity of AKT in T-cells by monitoring for the phosphorylation of the activation site at Ser473 (Fig. 2). For this, splenocytes from C57Bl6 mice were incubated at various times with anti-CD3 (1 ug/ml) or SC79 (4 ug/ml) followed by immunoblotting with anti-phospho-AKT or anti-AKT. A time course showed that SC79 induced phospho-AKT over an incubation period of 0-15 min (Fig. 2a). Further, the level of pSer473 induced by SC79 was comparable to that seen with anti-CD3 ligation, while the combination induced phosphorylation at levels that were similar to each separately (Fig. 2b). Uncut blots are seen in Fig. S2. These data showed that SC79 could induce the phospho-activation of AKT in T-cells at levels seen with anti-CD3 ligation.

### AKT activation decreases the presence CD4+FoxP3+ TILs

**Figure 3:**
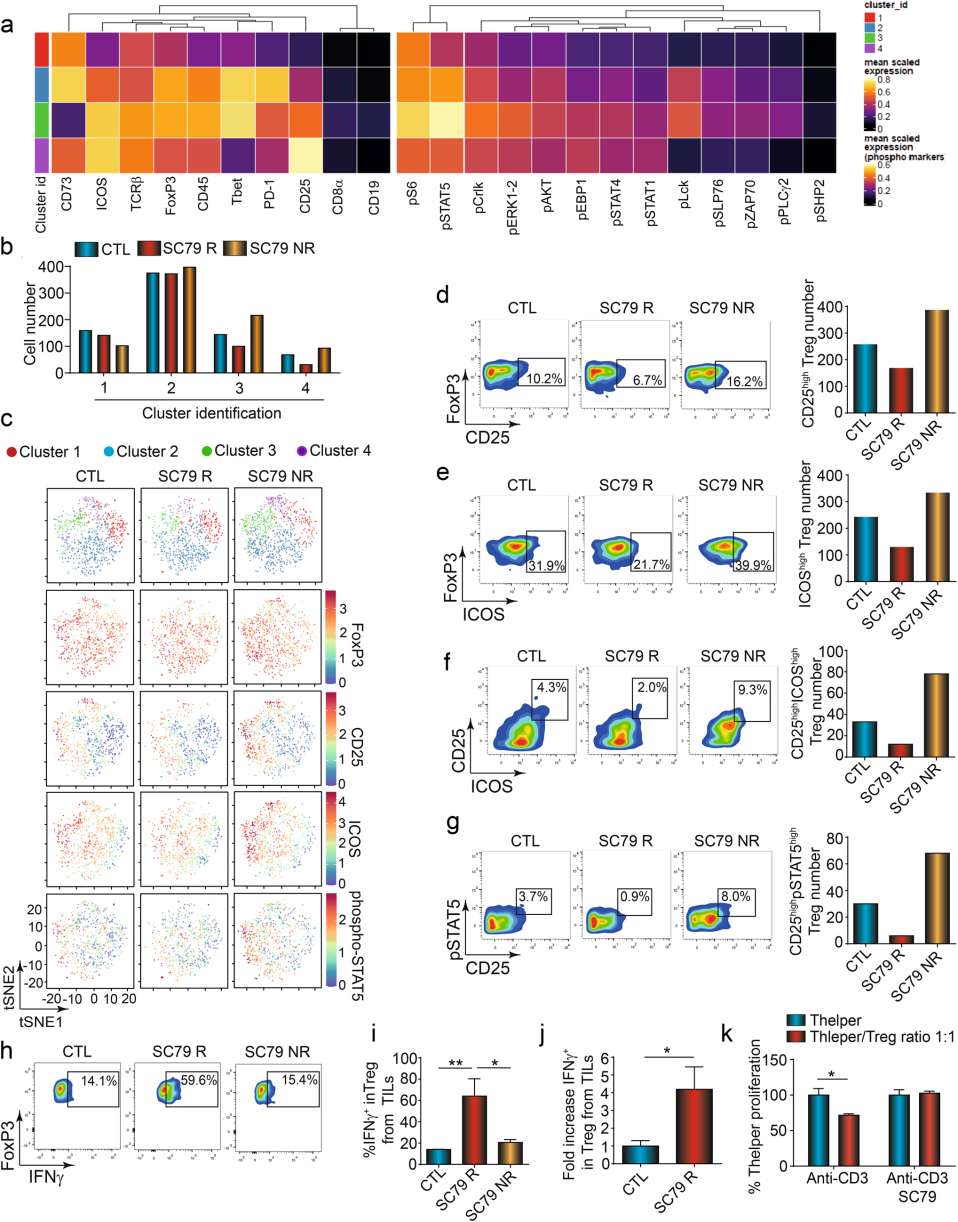
SC79 therapy decreases Treg infiltration with an increase in IFN-γ expression on CD4 Treg TILs. (**a**) Heat map derived from FlowSOM/UMAP analysis of CD4+ TILs (n = 3). (**b**) Histogram showing the per cent of cells in each cluster as seen in (**c**) involving tSNE analysis of TILs. **c**, tSNE analysis of TILs. (**d**) Analytical FACS profiles showing the frequency FoxP3 x CD25 expression (left panel). Histogram showing the numbers of CD25^hi^ Tregs in the TIL population (right panel) (n = 3). (**e**) Analytical FACS profiles showing the frequency of FoxP3 x ICOS expression, Histogram showing the numbers of ICOS^hi^ Tregs (right panel) (n = 3). (**f**) FACS profiles showing the frequency of CD25 x ICOS cells in the TIL population. Histogram showing the numbers of CD25^hi^/ICOS^hi^ Tregs (right panel). (**g**) Profiles showing the pSTAT5 x CD25 expression in the TIL population. Histogram showing the numbers of pSTAT5^hi^ x CD25^hi^ Tregs (right panel) (n = 3). (**h**) Profiles showing the frequency of FoxP3+ cells expressing IFN-γ in TIL populations. responder and non-responder tumors (n = 3). Analysis of Treg expressing IFN-γ frequency from CTL (n = 4), SC79 R (n = 3) and SC79 NR (n = 3) TILs, **i**, Percent of IFN-γ Tregs in the TIL populations. The percentage increased in responder but not non-responder tumors. **j**, Fold increase of Treg expressing IFN-γ from TILs after 48 h of in vitro activation with SC79 (n = 3). (**k**) SC79 reverses the ability of T-cells to suppress anti-CD3 proliferation as measured by an increase in the presence of SC79 after 72 h of culture (n = 3). Results from the figure (**a**) to (**g**) were obtained after CyTOF acquisition from a pool of 3 tumors in each group. For statistical analyses, parametric unpaired t-tests and one-way ANOVA were used. *p < 0.05, **p < 0.01. The R package CATALYST v1.12.1 was used to perform unsupervised clustering, and generate the heatmaps and tSNE plots. https://bioconductor.org/packages/release/bioc/html/CATALYST.html. https://f1000research.com/articles/6-748.

We next performed higher-order mass cytometry time of flight CyTOF analysis of CD4 and CD8+ TILs (Fig. 3). The gating strategy is shown in Fig. S3a. Firstly, CD4+CD8− TCRβ+ T-cells could be divided into subsets as visualised in a Heat Map (Fig. 3a) and by tSNE analysis (Fig. 3b,c). This analysis showed that FoxP3+ Treg clusters 3 and 4 were reduced in responder tumors. Cluster 3 corresponded to a FoxP3^hi^, CD25^int^, ICOS^hi^, T-bet^hi^, PD1^int^ population, while cluster 4 expressed FoxP3^int^, CD25^hi^, ICOS^hi^, T-bet^lo^ PD1^int^. Both reduced subsets shared a high-intermediate expression level of FoxP3, CD25 and ICOS, key markers of Tregs with suppressor function. ICOS has been reported to promote the generation, proliferation, and survival of Tregs^34,35^. Similarly, CD25 provides survival and proliferative signals in T-cells^36^, while PD-1 is an activation marker when expressed at intermediate levels^37^. In terms of signalling, both clusters had intermediate to high levels of pS6 and pSTAT5 as well as pCrkl, pERK1,2 and pAKT. Each of these is induced during the activation of T-cells^17^. Further, Cluster 3 could be distinguished from cluster 4 by its expression of phosphorylated (Y-505) pLck^18^.

Cytometric analysis of samples further confirmed the reduction in the per cent of FoxP3^+^CD25^+^ T-cells from 16.2% in SC79 NR to 6.7% of TILs in SC79 R mice (Fig. 3d, left panel). We also observed a decrease in the number of CD25^high^ Treg cells in SC79 R mice relative to the numbers of cells seen in the control and SC79 NR mice (i.e., per 1,000 CD45+ TILs) (right panel). In addition, cytometry confirmed the decline of FoxP3^+^ICOS^+^ and CD25^+^ICOS^+^ cells (Fig. 3e,f, respectively). This was seen in terms of the per cent of cells within the CD45+ cells (left panels) and in the numbers of cells (right histogram). Again, ICOS promotes the function and expansion of Tregs^34^. In this context, since CD25 signals via the transcription factor STAT5^36^, we also examined the expression of phospho-STAT5 in TILs (Fig. 3g). Indeed, we found that the reduction in CD25 expression was accompanied by a marked decrease in STAT5 phosphorylation, both in terms of percentage of cells (i.e., 8.0% SC79 NR to 0.9% SC79 R) and in cell number (left and right panels).

Importantly, at the same time, we observed a marked increase in the expression of the effector cytokine, interferon-γ (IFNγ) on the Tregs of SC79-treated mice (Fig. 3h–j). In one characteristic experiment, IFN-γ expression increased from 14.1 to 59.6 per cent of FoxP3+ TILs in responder mice (Fig. 3h). Importantly, no increase was seen in the non-responder tumors. Based on 3 experiments, we observed an increase in the percentage of Tregs TILs expressing IFN-γ+ from 16% in controls to a remarkable 63% of TILs from SC79 R mice (Fig. 3i). By contrast, CD4+Treg+ TILs from SC79 NR mice showed no increase in expression. In this vein, the expression of IFNγ could differentiate responder from non-responder mice. The overall increase was also observed when expressed as a fold-increase in CD4+Treg+ TILs in response to SC79 treatment (Fig. 3j).

Importantly, IFN-γ expression on Tregs has been reported to represent a phenotypic conversion to CD4^+^Th1-like cells with reduced suppressive activity^38^. We, therefore, assessed suppressor function of ex vivo SC79 treatment of isolated TILs from control mice (Fig. 3k). While Tregs from control mice inhibited anti-CD3 induced proliferation at a Teff/Treg ratio of 1:1, TILs incubated with SC79 failed to suppress T-cell proliferation. Lastly, SC79 also increased in the expression of IFN-γ on CD4+ FoxP3− cells (i.e., 8.1% to 39.5%) (Fig. S4a) in a manner that could distinguish responsive from non-responsive mice (Fig. S4b).

Overall, these data show that SC79-mediated AKT activation can reduce the presence of suppressive Tregs, in a process that may involve the conversion of Tregs to IFN-γ+ CD4^+^Th1-like T-cells. Further, IFN-γ expression was the major distinguishing feature of SC79-induced AKT activation on CD4+P3+ and CD4+ FoxP3− TILs which could distinguish responsive from non-responsive mice.

### AKT activation causes a major increase in CD8+ TILs

**Figure 4:**
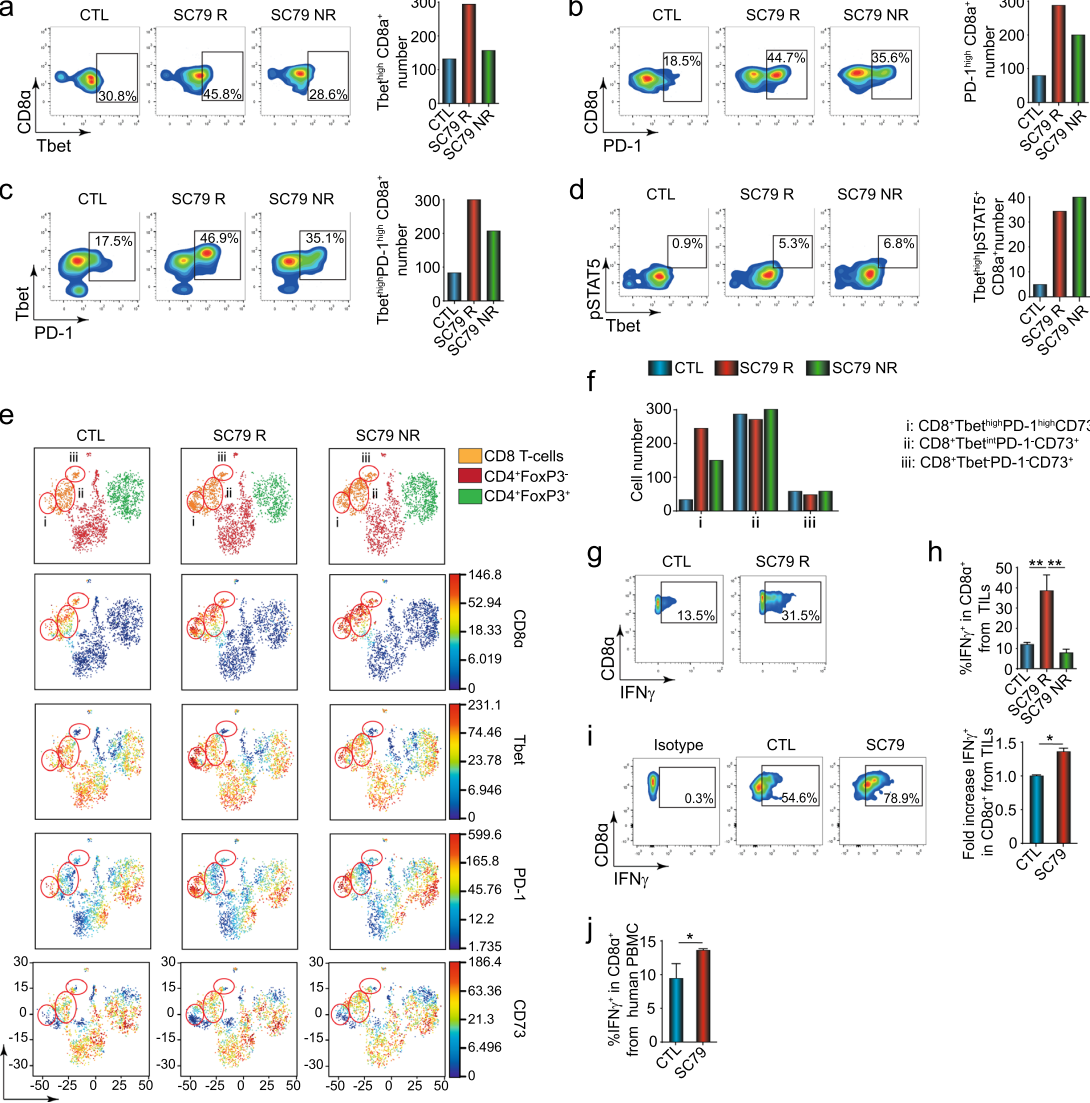
SC79 increases tumor infiltration of CD8-T cells and CD8 T-cells expressing IFNγ. (**a**) Profiles showing an increase in the per cent of CD8alpha x Tbet expression in responsive mice (left panel). Histogram showing an increase in numbers of CD8alpha x Tbet TILs responder mice (right panel) (n = 3). (**b**) Profiles show an increase in the per cent of CD8alpha x PD-1 expression (left panel). Histogram showing the increase in numbers of CD8alpha x PD-1 TILs (right panel) (n = 3). (**c**) FACS profiles show an increase in the per cent of Tbet x PD-1 expression (left panel). Histogram showing the increase in numbers of Tbet x PD-1 TILs (right panel). (**d**) Profiles showing an increase in the per cent of pSTAT5 x Tbet expression (left panel). Histogram showing the increase in numbers of pSTAT5 x Tbet 1 TILs (right panel) (n = 3). (**e**) viSNE analysis of TILs with a focus on CD8+ T-cells (circled). Subpopulations of CD8 T-cells were obtained by the expression of Tbet, PD-1, and CD73 (n = 3). (**f**) Histogram showing the frequency and cell number of CD8 T-cell subpopulations outlined in Fig. 2e. (**g**) Profiles showing the frequency of CD8 T-cell TILs expressing IFN-γ. (**h**) Histogram showing the increase in the percentage of CD8 T-cells expressing IFN-γ from CTL (n = 4), SC79 R (n = 3) and SC79 NR (n = 3) mice. An increase in CD8+ IFN-γ+ TILs was seen in responder but not non-responder mice. **i,** IFN-γ expression on CD8 T-cells is increased by treating ex vivo TILs with SC79 from control mice. TILs from untreated mice were cultivated in a 96-well plate for 3 days with anti-CD3 with recombinant IL-2 in the presence or absence of SC79. While 54.6 per cent of CD8+ control cells showed IFN-γ expression, this increased further to 78.9 per cent due to culturing in SC79 (n = 3). **j,** IFN-γ expression in CD8 T-cell from a healthy donor after 48 h of culture with or without SC79 (n = 4). Results from the figure (**a**) to (**g**) were obtained after CyTOF acquisition from a pool of 3 tumors of each group. For statistical analyses, one-way ANOVA and a non-parametric unpaired t-test were used. *p < 0.05, **p < 0.01. The R package CATALYST v1.12.1 was used to perform unsupervised clustering, and generate the heatmaps and tSNE plots. https://bioconductor.org/packages/release/bioc/html/CATALYST.html. https://f1000research.com/articles/6-748.

As mentioned, we observed an increase in the presence of CD8+ T-cells in tumors from mice treated with SC79 (Fig. 1j). In exploring the phenotype of the CD8 TIL subset in greater depth, we observed an increase in the presence of CD8+ T-bet^hi^ TILs from responsive, but not non-responsive, mice (i.e., from 30.8% in control to 45.8% in SC79R samples and 28.6% in NR samples) (Fig. 4a, left panels). An increase in the numbers of T-bet^hi^CD8+ T-cells in tumors was also seen in responsive, but not non-responsive mice (right panels). Further, we observed an increase in PD-1^hi^ CD8+ TILs in terms of % expression and numbers of TILs expressing PD-1 (Fig. 4b, left and right panels) and in CD8+ T-cells co-expressing T-bet and PD-1 in SC79 treated mice (Fig. 4c, left and right panels). In this case, a partial increase in PD-1 expression was seen in non-responder mice showing that some TIL activation occurred. Similarly, an increase in the co-expression of T-bet^hi^ and pSTAT5 was seen in both groups (Fig. 4d).

viSNE analysis further identified three clusters within the CD8^+^ TIL population (Fig. 4e). In this case, we observed an increase in a cluster defined by the group expression of CD8^+^T-bet^high^PD-1^high^CD73^-^ in SC79 responder mice (i.e., cluster i) (Fig. 4f). A partial increase in the presence of the same cluster was observed in non-responding tumors. Interestingly, neither of the CD8 subsets expressing the inhibitory receptor CD73 showed an increase^39^.

Importantly as well, SC79 therapy further increased the expression of IFN-γ amongst CD8^+^ TILs (i.e., 13.5 for control to 31.5 per cent in responder tumors) (Fig. 4g; also see separate experiment Fig. S5). Based on the concatenated data from 3 experiments, we observed a significant increase in CD8^+^ TILs expressing IFN-γ in responder mice, but not in NR mice (i.e., 12 for control to 28 per cent in responder tumors) (Fig. 4h). These data showed that IFN-γ expression on CD8^+^ TILs could distinguish responder from non-responder mice, as previously outlined for IFN-γ expressing CD4^+^ TILs.

Similarly, viSNE analysis on gated CD8+ TIL subset confirmed these findings (Fig. S6). In this case, as shown by viSNE and in the Heat Maps, three clusters were identified where cluster 1 expressing T-bet and PD-1 showed the greatest increase (Fig. S6a,c). The subset also showed an increase in pLck, and to a lesser degree, pERK, pAKT, pCrkL and pSTATs 5 and 4. Remarkably, Cluster 1 was also defined by the markedly lower level of CD73 expression (Fig. S6c–j).

Further, we conducted ex vivo experiments with TILs isolated from control mice (Fig. 4i). We showed that we could re-capitulate the results with an increase in IFN-γ expression on CD8 TILs by incubating with SC79. TILs from untreated mice were cultivated in a 96-well plate for 3 days with anti-CD3 with recombinant IL-2 in the presence or absence of SC79 as outlined in the *Methods*. While 54.6 per cent of CD8+ control cells showed IFN-γ expression, this increased further to 78.9 per cent due to culturing with SC79.

Similarly, by using human peripheral T-cells from healthy donors (Fig. 4j). PBLs were also cultivated with anti-CD3 and anti-CD28 with recombinant human IL-2 with or without SC79 for 3 days. In this case, a lower level of IFN-γ expression on CD8 T-cells was observed; however, this level was significantly increased by culture with SC79. Collectively, these data show that the chemical activation of AKT increases the expression of IFN-γ on CD4+ Tregs and CD8+ T-cells.

Lastly, we assessed whether SC79 therapy could affect stages of differential (Fig. S7). TILs from untreated and SC79 treated tumors were stained to identify classic CD44+ CD62L− (effector memory) or CD44+CD62L+ (central memory) subsets. We found no difference in the two TIL subsets between control and SC79-treated mice.

### AKT activation induces different phosphorylation events in CD4+FoxP3+ and CD8+ subsets TIL subsets

**Figure 5:**
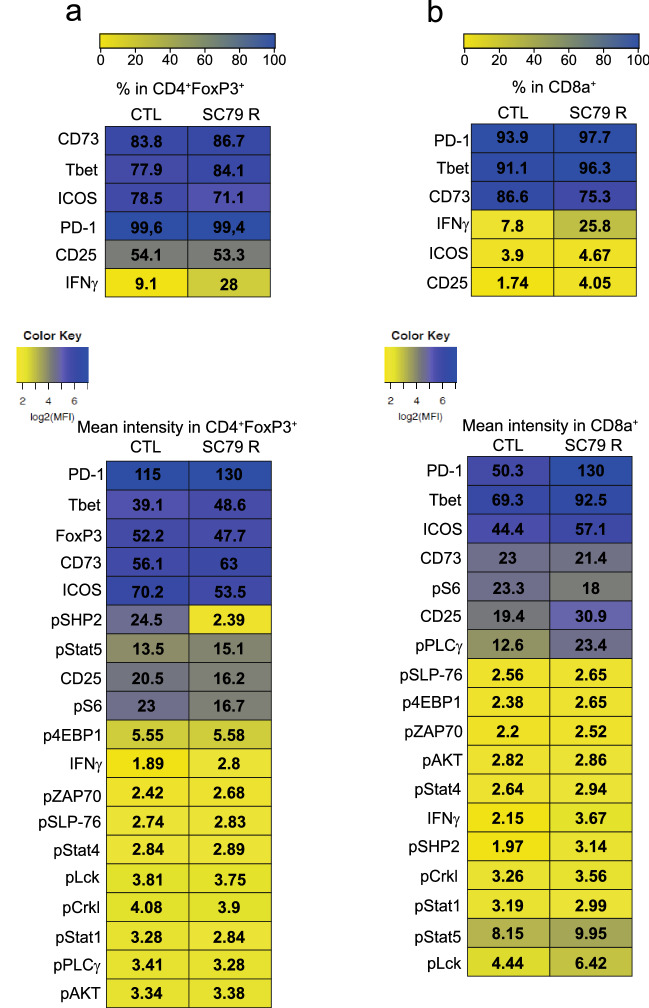
CyTOF analysis shows phosphorylation AKT mediated signalling events in different TIL subsets. (**a**) Patterns in CD4+FoxP3 TILs (n = 3). Upper panel: % representation in CD4+FoxP3+ subset. lower panel: MFI representation in CD4+FoxP3+ subset (* denotes noteworthy changes). CD4+ FoxP3−, CD4+FoxP3+ and CD8+ subsets showed quantitative similarities and differences in levels of different phospho-proteins. pSHP2 was expressed at the highest level in CD4+FoxP3+ Tregs where it underwent a striking tenfold decrease in response to SC79 therapy (from 24.5 to 2.39). (**b**) Patterns in CD8+ TILs. Upper panel: % representation in CD8+ subset. lower panel: MFI representation in CD8+ subset. * denotes noteworthy changes in pPLC-γ most affected in CD8+ T-cells where it increased from 12.6 to 23.4, an effect not seen in CD4+ FoxP3− or + T-cells. Results were obtained after CyTOF acquisition from a pool of 3 tumors of each group.Heat maps were generated with Morpheus, https://software.broadinstitute.org/morpheus The uploaded information was simply performed in order obtain a colored chart. There was no statistical analysis performed by the Morpheus site for this purpose. For this reason, no version number or name was provided by the website. The loaded data for colored chart formation was conducted on the website in March 2019. “The log2 values were obtained with CATALYST https://www.bioconductor.org/packages/devel/bioc/manuals/CATALYST/man/CATALYST.pdf”.

CyTOF analysis of phosphorylation signalling events also revealed specific changes in the activation of signalling proteins induced by AKT activation in the CD4+FoxP3+ and CD8+ TIL subsets (Fig. 5a,b; see highlights with asterix). In terms of percentages of cells, as mentioned, we observed primarily an increase in the % of CD4+ Tregs and CD8+ T-cells expressing IFN-γ. The contrasted with the MFI for IFN-γ expression which did not show a change on either CD4 or CD8+ cells. A greater percent of CD4+FoxP3+ cells expressed Tbet. We also observed a decrease in the % of CD8+ T-cells expressing the inhibitory receptor CD73. In terms of the MFI of expression, we observed major increases in the expression of expression levels of PD1 and Tbet in both subsets of cells. A moderate increase in the expression of CD25 was also observed in the CD8 population, consistent with its increased presence in tumors following treatment with SC79. Interesting, pSHP2 was expressed at the highest level in CD4+FoxP3+ Tregs where it underwent a striking tenfold decrease in response to SC79 therapy (from 24.5 to 2.39). This change in pSHP2 was interesting since PD-1 binding to SHP-2 is thought to be responsible for its negative signalling in cells^23^. By contrast, in CD8+ T-cells, pPLC-γ was most affected with an increase from 12.6 to 23.4, an effect not seen in CD4+ FoxP3− or + T-cells. This is also consistent with the expanded presence of CD8+ TILs since PLC-γ activation by binding to the adaptor LAT is essential for T-cell activation and expansion^40^. These data indicate that SC79 activation of AKT can affect different pathways in CD4 versus CD8 TIL subsets.

### SC79 activation of AKT and tumor regression is mediated via transcription factor T-bet

**Figure 6:**
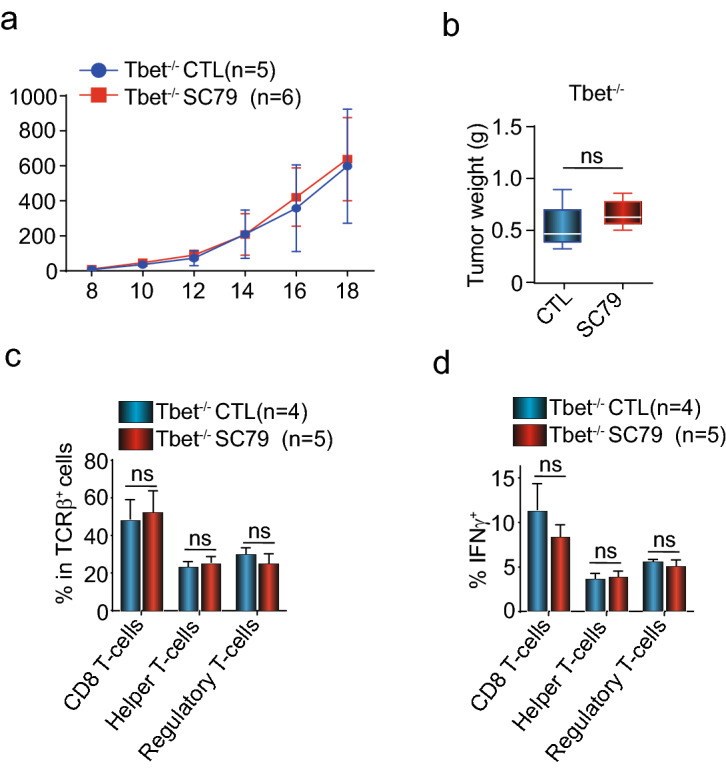
The transcription factor T-bet is essential for SC79 treatment efficiency. (**a**) T-bet is needed for the ability of SC79 to limit B16 tumor growth. No difference in tumor growth was seen in wild-type (*T-bet*+*/*+) versus *T-bet−/−* mice (n = 4). (**b**) T-bet is needed for the ability of SC79 to reduce tumor size. There was no difference in tumor weight in wild-type (*T-bet*+*/*+) versus *T-bet−/−* mice. Control (corn oil) or SC79 treated. (**c**) Frequency of CD8 T-cells, helper T-cells (CD4+ FoxP3−) and suppressive regulatory T-cells (Tregs) (CD4+FoxP3+) among TCRβ^+^ cells from wild-type (*T-bet*+*/*+) versus *T-bet−/−* mice. 79. (**d**) Frequency of IFNγ^+^ CD8 T-cells, helper T-cells (CD4+ FoxP3−) and suppressive regulatory T-cells (Tregs) (CD4+FoxP3+) among TCRβ^+^ cells from wild-type (*T-bet*+*/*+) versus *T-bet−/−* mice.

The transcription factor T-bet plays a central role in the generation of CD8+ effector T-cell responses^41^. It also regulates interferon γ expression^42,43^ and Treg function^44^. We, therefore, next assessed whether SC79 therapy could regress tumors in *T-bet*^*−/−*^ mice (Fig. 6a,b). With these mice, we found that SC79 was completely ineffective in reducing the growth and size of B16 tumors. It also prevented the increase in CD8+ T-cells and the loss of FoxP3+ Treg TILs (regulatory T-cells) normally induced by SC79 therapy (Fig. 6c). Further, it failed to support the SC79-induced increased expression of IFN-γ on CD4 and CD8^+^ TILs (Fig. 6d). These observations indicated that the ability of small molecule induced SC79 to stimulate the TIL response for tumor regression as well as in upregulating IFN-γ expression is dependent on the transcription factor T-bet.

### Modelling of SC79 binding to AKT

The original study by Jo and co-workers modelled the binding of the small molecule to the SC79 to the PH domain of AKT1^32^. To confirm and extend these findings, we conducted our own drug-protein modelling studies between SC79 and AKTs and could confirm that SC79 binds to the interface of the PH and kinase domains of the isoforms of AKT-1, as well as AKT-2 and 3 (Fig. S8, Table S1). SC79 was found to bind on the interface between 6S9W (AKT1) and PH domain (1UNR) of AKT with a binding affinity estimated at − 7.0 kcal/mol. The interaction is stabilized through five conventional hydrogen bonds (i.e., residues GLU17, TYR18, ILE19, ARG25, and GLN390), Van der Waals bonds (i.e., residues ARG23, LYS14, LYS385, SER381, GLY382, LEU392) carbon-hydrogen interaction (i.e., GLY16), and Halogen bond interaction on SER378. A more complete description of our analysis is summarized in SFig. 8g; Table S1). Our findings suggest that the SC79 should show a gradient of effectiveness according to the docking scores of AKT2 > AKT1 > AKT3.

## Discussion

While anti-PD-1 blockade has achieved impressive survival rates for certain cancers, most patients are not cured, underscoring the need for alternate or complementary clinical interventions. In this context, we and others have shown that TCR and CD28 cooperatively activate AKT in T-cells^22^, while PD-1 inhibits its activation^23^. Here, we have queried whether the direct small-molecule activation of AKT can activate the immune system to limit the growth of tumors that are resistant to anti-PD-1 blockade. In this way, one would be substituting at the level of a small molecule, a key signal that is normally activated with anti-PD-1 immunotherapy. The assumption is that in certain tumors, the resistance might be associated with a defect in the ability of PD-1 blockade to promote the activation of AKT. Most clinical reagents in use against AKT are inhibitors^45^. SC79 is the first reported small-molecule activator of AKT but has not been tested in tumor models^33^. In this study, we show for the first time that small-molecule activation of AKT limits the growth of a B16 melanoma and EMT-6 breast cancer that are poorly responsive to anti-PD-1 therapy. This, in the case of the B16 tumor model, was accompanied by an increase in CD8+ TILs, concurrent with a loss of suppressive CD4+ Tregs TILs. Further, we found that a hallmark of AKT activation in T-cells in tumors was the induction of a major increase in the effector cytokine, IFN-γ in the CD4 and CD8 cells. Further, the increase in IFN-γ expression distinguished TILs in tumors that were responsive to SC79 therapy from non-responsive tumors. Our findings indicate AKT activation by a small-molecule (AKT activation therapy) as a novel potential approach that can augment CD8 responses and deplete Tregs in overcoming resistance to anti-PD-1 therapy.

The activating effect of SC79 allowed us to examine the role of AKT activation in tumor immunity and to explore the therapeutic potential of this small molecule. The induced increase in CD8 TILs and reduction of Tregs were both found in responsive tumors and was not seen in non-responsive tumors. Similarly, the increase in IFN-γ expression in CD4+ Tregs and CD8 TILs was significantly correlated with the ability of SC79 to limit tumor growth. This clearly showed that AKT activation induces IFN-γ expression on CD4 and CD8 T-cells in a manner correlated with tumor rejection. In this context, IFN-γ is an effector cytokine critical for both innate and adaptive immunity. It activates macrophages and induces the expression of class II antigens of the major histocompatibility complex on antigen-presenting cells. Although the effects of SC79 on Tregs have been reported in autoimmunity^46^, a direct connection of AKT activation in T-cells to the induction of IFN-γ in tumors has not been reported.

We first found that AKT activation therapy reduced the presence of suppressive effector CD4+ Treg T-cells in tumors. This offers an alternative small molecule approach for the reduced presence of Tregs in tumors. There was little detectable effect on the drug on the in vitro growth of the B16 cells, which although this does not completely exclude direct possible effects, it suggests that the main mechanism of restricted tumor growth is due to the effects on the immune system. Effects on Treg depletion in tumors has previously been documented with anti-CTLA-4 and anti-CD25 biologic therapy where depletion occurred via ADCC^2,14,47^. In this case, the effectiveness of anti-CTLA-4 varied with the expression status of CTLA-4 on effector CD8+ T cells^2,14,48^ and appeared less effective in human cancer trials^2,14^. Further, our CyTOF data showed the preferential loss of Treg TILs co-expressing CD25 and ICOS. CD25 promotes the expansion of effector Tregs, while CD4+FoxP3+ ICOS^+^ are also more suppressive than ICOS- Tregs^34,36^. This small-molecule approach, therefore, offers a potential alternative way to deplete Tregs, especially the more suppressive ICOS+ effector Treg TILs. Consistent with this finding of an inhibitory role of AKT activation on Tregs, the retroviral overexpression of AKT can block Foxp3^+^ Treg cell development^49^.

In addition, our study showed that in inducing IFN-γ expression, SC79 therapy promoted the conversion of Tregs to an IFNγ+ Th1-like cell. In addition to promoting the appearance of CD4+FoxP3+ IFNγ+ Th1-like TILs, we found that SC79 ex vivo treated TILs also increased IFNγ+ expression, and further, the treated cells had reduced suppressor activity on the anti-CD3 induced proliferation of T-cells. The effect of SC79 on Treg plasticity had previously been reported in autoimmunity^46^, but not in the case of TILs in cancer. It is also consistent with its role of upstream in the PI 3K pathway in regulating this plasticity^50^. However, reports documenting this transition generally find that FoxP3 expression is conserved. In our case, we observed both the loss of FoxP3 expressing Tregs and the acquisition of IFN-γ expression with reduced suppressor function. It may therefore be that AKT activation acts to both limit FoxP3 TILs and facilitate the Th1-like helper transition in the TIL population. To our knowledge, our findings are one of the first examples of a small-molecule therapeutic approach that depletes Tregs and converts FoxP3+ Tregs into Th1-like cells in the tumor microenvironment (TME). A small molecule inhibitor of the MALT1 protease in the CARMA1-BCL10- MALT1 (CBM) complex is also involved the conversion of Tregs to IFN-γ producing cells^51^.

In the case of glioblastoma, anti-PD-1 treatment shifts the profile of Tregs in vivo to more IFN-γ production^52^. In the case of tumors that are less responsive to anti-PD-1, the use of SC79 can provide a similar potential outcome. In terms of signalling, CyTOF analysis revealed that pSHP2 underwent a striking decrease preferentially in Tregs, a signalling protein that it can generate general negative signals in T-cells^23^. This suggests the possibility that pSHP2 may be important in sustaining the presence of CD4+FoxP3+ Tregs in tumors. SHP-2 is also thought to contribute to negative signalling by PD-1^23^. It remains to be determined whether the unexpected effect of AKT activation on SHP2 is responsible for the loss of Tregs or their conversion to Th1-like cells in the TME.

Lastly, we observed that SC79 therapy caused a major increase in CD8+ TILs expressing IFN-γ. IFN-γ is a surrogate marker for effective cytotoxic T lymphocytes (CTL)^53^. Further, anti-PD-1 therapy requires IFN-γ production^54^. SC79 induced a higher percentage of CD8+ T-cells expressing IFN-γ than we observed with anti-PD-1 blockade. Further, with CyTOF analysis, we showed a specific increase in CD8+ TILs expressing the transcription factor T-bet and the co-receptor, PD-1^+^ on TILs. Potentially, SC79 activation of AKT and its upregulation of T-bet could contribute to the upregulation of IFN-γ since the transcription factor has previously been shown to regulate interferon γ production^42,43^. T-bet is also needed for the generation of the CD8^+^ cytotoxic effector cell where T-bet deficient mice show diminished cytotoxicity^42^. IFN-γ expression is induced by signals from the T cell receptor (TCR) and the IFN-γ receptor itself^55,56^. In this sense, the increased production of IFN-γ could re-enforce the production of IFN-γ via binding to its receptor. In terms of signalling, we observed that SC79 increased the phosphorylation of PLC-γ selectively in CD8+ T-cells consistent with the essential role of this mediator in T-cell activation. Intriguingly, these data demonstrate that AKT regulates different pathways in different subsets of T-cells. We also observed a moderate increase in the expression of PD1, a marker for T-cell activation. Moreover, SC79 increased CD8+ TILs with the higher levels of T-bet and PD-1 but lacking the expression of CD73, a surface receptor that can inhibit T-cell function via adenosine receptor (AR) signaling^57^. This inhibitory receptor would otherwise limit proliferation^58^. Finally, our own docking analysis confirmed the binding of SC79 to the PH domain of all isoforms of AKT (1,2,3). Overall, our study identifies a novel potential therapeutic approach using a small-molecule activator to bypass the need for ICB and induce novel T-cell responses against tumors.

## Materials and methods

### Mice, B16 cell implantation and treatments

C57Bl/6 and T-bet^−/−^ were bred in our animal facility (Charles River). All animal experiments were approved with the animal committee agreement of the Research Center of Hospital Rosemont Maisonneuve, Montréal, Canada. Project Number 2021–2525. All experimental protocols were approved by the institutional committee, Comité de protection des animaux CIUSSS de l'Est-de-l'Île-de-Montréal. All methods were carried out following relevant guidelines and regulations. The study was also carried out in compliance with ARRIVE guidelines. All methods were also carried out following relevant guidelines and regulations apart from ARRIVE guidelines. B16 cells, obtained as a kind gift from Dr Nathalie Labrecque (CR-HMR), were cultivated in DMEM complemented with 5% heat-inactivated FBS (Invitrogen). Before implantation, mice were shaved on a flank. Then, they received a subcutaneous injection of 50,000 B16 cells per mouse. On day 7 after implantation, mice were randomized and treatments were started. SC79 (ethyl 2-amino-6-chloro-4-(1-cyano-2-ethoxy-2-oxoethyl)-4*H*-chromene-3-carboxylate) (purchased from (Selleckchem) was diluted in corn oil and mice received 0.4 mg of the drug in a volume of 200 µl every two days. Control mice received 200 µl of corn oil every two days. About anti-PD-1 (clone: J43), antibodies were diluted in PBS and mice received 200 µg of antibodies in a final volume of 200 µl every 3 days.

### CyTOF mass cytometry

Tumor infiltrated lymphocytes were incubated with cisplatin (Fluidigm) for viability for 5 min at room temperature. Incubation was stopped with MaxPar Cell Staining Buffer (Fluidigm), cells were centrifugated at 300 g for 5 min at 4 °C and washed with MaxPar Cell Staining Buffer. Then, cells were directly fixed with cold TFP Fix/Perm Buffer (Fluidigm) for 50 min at 4 °C. Fixation was stopped by adding cold TFP Perm Wash Buffer and washing in TFP Perm Wash Buffer. Cells were frozen in FBS-7%DMSO and stored at -80 °C. The day before the acquisition, cells were slowly defrosted on ice and washed in TFP Fix/Perm Wash Buffer. After, cells were incubated for 20 min in chilled Perm Buffer III (Beckton Dickinson). Cells were washed in TFP Fix/Perm Wash Buffer and incubated for 10 min at room temperature with Fc block (BioLegend). Then, cells were stained with all the antibodies, diluted in TFP Perm Wash Buffer, for 20 min at 4 °C. Cells were washed in TFP Perm Wash Buffer and incubated overnight with Cell-Id intercalator -Ir (Fluidigm) diluted in MaxPar Fix/Perm Buffer (Fluidigm). Finally, cells were washed in MaxPar Staining Buffer, resuspended in MaxPar Cell Acquisition Buffer (Fluidigm) and acquired using CyTOF System Helios (Fluidigm,). Classical analyses were performed using FlowJo software (Beckton Dickinson), Cytobank (Beckman Coulter) for viSNE analyses and FlowSOM analysis was performed with ConsensusClusterPlus (Bioconductor).

Concerning CyTOF staining all the products were purchased from Fluidigm, South San Franscico, CA. The following antibodies were used: 89Y-conjugated anti-CD45 (clone: 30-F11), 169Tm-conjugated anti-TCRβ (clone: H57-597), 145Nd-conjugated anti-CD4 (clone: RM4-5), 146Nd-conjugated anti CD8α (clone: 53–6.7), 159 Tb-conjugated anti-PD-1 (clone: 29F.1A12), 161Dy-conjugated anti-Tbet (clone: 4B10), 158Gd-conjugated anti-FoxP3 (clone: FJKK-16s, 176Yb-conjugated anti-ICOS (clone: 7E.17G9), 151Eu-conjugated anti-CD25 (clone: 3C7); 165Ho-conjugated anti-IFN-γ (clone: XMG1.2), 150Nd-conjugated anti-pStat5 (clone: 47); 154 Sm-conjugated anti- CD73 (clone: TY/11.8); 159 Tb-conjugated anti-CD279/ PD-1 (clone: 29F.1A12); 141Pr-conjugated anti-pSHP2 [Y580] (clone: D66F10); 172Yb-conjugated anti-pS6 [S235/ S236] (clone: N7-548), 149Sm-conjugated anti-p4E-BP1 [T37/ T46] (clone: 236B4); 171Yb-conjugated anti-pZAP70 [Y319]/pSyk [Y352] (clone: 17a); 156Gd-conjugated anti-pSLP-76 [Y128] (clone: J141- 668.36.58); 148Nd-conjugated anti-pStat4 [Y693] (clone: 38/p-Stat4); 143Nd-conjugated anti-pCrkl [Y207] (polyclonal); 153Eu-conjugated anti-pStat1 [Y701] (clone: 4a); 144Nd-conjugated anti- pPLCg2 [Y759] (clone: K86-689.37); 152Sm-conjugated pAkt [S473] (clone: D9E) and 162Dy-conjugated anti-pLck (clone: 4/Lck-Y505). For the FACS staining, the following antibodies were used: PerCP-Cy5.5-conjugated anti-CD45 (clone: 30-F11), BV605-conjugated anti-CD4 (clone: RM4-5), BV650-conjugated anti-CD8 (clone: 53–6.7), eFluor450-conjugated anti-FoxP3 (clone: FJK-16s), and APC-conjugated anti-IFN-γ (clone: XMG1.2,). All the antibodies used for CyTOF were diluted in 1/100 as recommended by the Fluidigm company. About the the antibodies for flow cytometry, all the antibodies for surface marker were diluted in 1/50 and for the intracellular/nuclear markers in 2/50.

### Flow cytometry

For general flow cytometry analysis, tumor infiltrated lymphocytes were resuspended in PBS to be incubated with Fixable Viability Stain 510 (Beckton Dickinson) for 20 min, in dark and at 4 °C. Then, cells were washed in FACS Buffer and incubated for 20 min at 4 °C and in the dark with membrane antibody staining (CD45, CD4 and CD8) or corresponding isotype controls diluted in FACS Buffer. Cells were washed in FACS Buffer and fixed for 45 min, at 4°Cin dark, with Fixation/Permeabilization Buffer (Invitrogen). After, cells were washed with Permeabilization Buffer (Invitrogen) and incubated for 30 min at 4 °C in dark with intracellular/intranuclear antibodies staining or corresponding isotype controls beforehand diluted in Permeabilization Buffer. Finally, cells were washed with Permeabilization Buffer and resuspended in FACS Buffer. The acquisition was performed using BD LSRFortessa X-20 and DIVA software (Beckton Dickinson). FACS analyses were performed using FlowJo software.

### Tumor infiltrated lymphocyte culture

TILs were cultivated in a 96-well plate for 3 days with anti-CD3 at 5ug/ml (clone: 2C11), recombinant mouse IL-2 at 20U/ml (StemCell Technologies) and with or without SC79 at 4ug/ml. Golgi Stop was added 4 h before the end of the culture. Then, cells were stained for CD4 and CD8 in FACS buffer, fixed with Fixation/Permeabilization Buffer for 45 min and stained for FoxP3 and IFN-γ in Permeabilization Buffer.

### Extraction, culture and FACS staining of peripheral blood mononuclear cells

Blood was collected from a healthy donor leukoreduction system chamber (Hema Quebec). Peripheral blood mononuclear cells (PBMCs) were freshly isolated by dextran sedimentation (Corning, Corning, NY). Then, PBMC were cultivated for 3 days with anti-CD3 at 5ug/ml (clone: OKT3), anti-CD28 at 2ug/ml (clone: 9.3), recombinant human IL-2 (StemCell Technologies) at 20U/ml and with or without SC79 at 4ug/ml. Golgi Stop was added 4 h before the end of the culture. Then, cells were resuspended in PBS to be incubated with Fixable Viability Stain 510 for 20 min, in dark and at 4 °C. After, they were stained with PercP Cy5.5-conjugated anti-CD3 (clone: UCHT1) and BV711-conjugated anti-CD8 (clone: RPA-T8) in FACS buffer for 20 min. After, cells were fixed with Fixation/Permeabilization Buffer for 40 min and finally stained for APC Cy7-conjugated anti-IFN-γ (clone: 4S.B3) during 30 min in Permeabilization Buffer.

### Suppression assay

CD4^+^CD25^-^ effector T (Teff) cells and CD4^+^CD25^+^ Treg cells were purified from the spleens C57Bl6 mice by using a CD4^+^CD25^+^ Regulatory T Cell Isolation Kit (Miltenyi). Teff cells were prelabeled with 5 mM CFSE (Invitrogen) for 10 min at 37 °C. CFSE-labeled Teff cells (1 × 10^5^) were cocultured in flat-bottomed 96-well plates with Treg cells (1 × 10^5^) (ratio 1:1) and antigen-presenting cells (1 × 10^5^) in RPMI 1640 with 10% FBS, 100 units/ml penicillin, 100 mg/ml streptomycin, 50 mM 2-mercaptoethanol, 1 M HEPES, and 5 μg/ml soluble anti-CD3 (clone: 2C11). Antigen-presenting cells that had been previously treated with mitomycin (50 μg/ml) for 45 min at 37 °C were added to the culture medium. Cells were then incubated at 37 °C in an atmosphere of 5% CO2. After 3 days of culture, the cells were stained with BV605-labeled anti-CD4 antibody (clone RM4-5) and the proliferation of Teff cells was determined by FACS.

### Molecular modeling

To confirm the binding of SC79 to AKT isoforms, we modelled the binding to AKT isoforms 1,2,3. The AKT model structures were obtained from the RCSB-Protein Data Bank (PDB) (https://www.rcsb.org). Autodock Tools software was initially used where water molecules were removed from each protein, polar hydrogen atoms were added and Gasteiger charge was assigned and then converted to (PDBQT) format. SC79 (ethyl 2-amino-6-chloro-4-(1-cyano-2-ethoxy-2-oxoethyl)-4*H*-chromene-3-carboxylate) (purchased from Sellecchem) and ATP structures was collected from the PubChem^56^. Autodock tools hydrogen atoms were added, required active torsions were assigned, the Gasteiger charge was computed then converted to pdbqt format. Protein–protein docking was performed using ZDOCK and HDOCK^59^**.** The result obtained from ZDOCK and HDOCK was then compared and the best complex model was derived according to Docking Score and RMSD (Root mean square deviation) (Å) where it was docked against SC79 and ATP. The docking of SC79 was then performed with all three complexes by using AutoDock Vina^60^. The docking of ATP was also performed with the resultant complexes by using the same software. In total, we performed blind docking of 6 complexes, first without ATP, then with ATP to see if the presence of ATP affects the interaction of SC79. Complexes were visualized and analysis resulted presented using Biovia Discovery studio 2020 and chimera 1.14 software. Data is not deposited since it is based on the virtual screening of pre-existing data.

### Statistics

All statistical analyses were performed using GraphPad Prism 6 software.

### Ethics approval

All animal experiments were conducted with the animal committee agreement of the Research Center of Hospital Rosemont Maisonneuve, Montréal, Canada. Project Number 2021–2525.

## Supplementary Information

Supplementary Information.

## Data Availability

There are no new sequences for proteins, DNA or RNA but any additional data will be available upon reasonable request. Any additional raw data for the paper can obtained from Drs Santinon (francoissantinon@gmail.com) or Rudd (christopher.e.rudd@umontreal.ca).
