# Supplementary material for: Direct AKT activation in tumor-infiltrating lymphocytes markedly increases interferon-γ (IFN-γ) for the regression of tumors resistant to PD-1 checkpoint blockade
Source: Sci Rep. 2022 Nov 2;12:18509. doi: 10.1038/s41598-022-23016-z (PMC9630443; doi:10.1038/s41598-022-23016-z)
Supplement: Supplementary file 1 — Supplementary Information. [file 41598_2022_23016_MOESM1_ESM.pptx]

## Slide 1
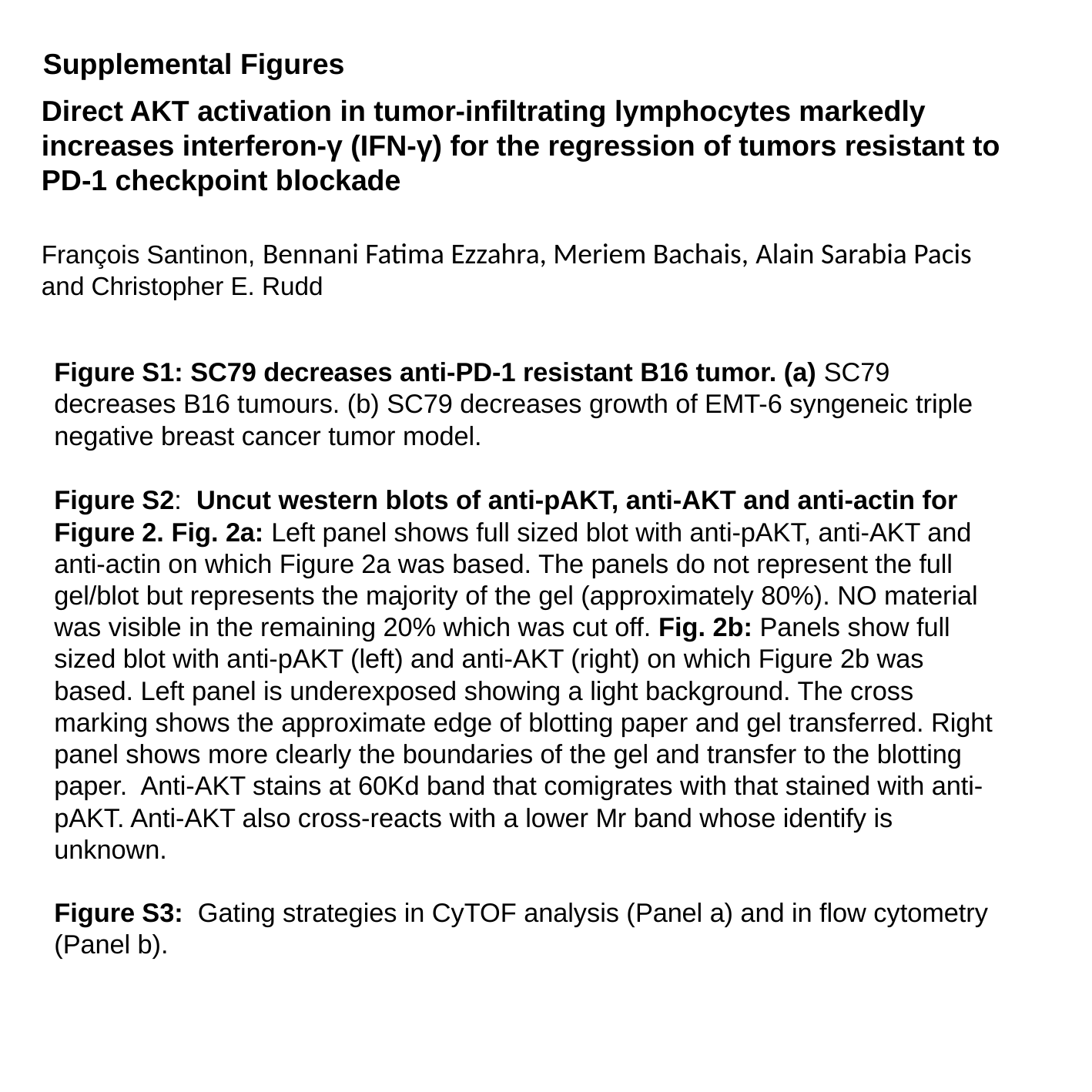

Supplemental Figures
Direct AKT activation in tumor-infiltrating lymphocytes markedly increases interferon-γ (IFN-γ) for the regression of tumors resistant to PD-1 checkpoint blockade
François Santinon, Bennani Fatima Ezzahra, Meriem Bachais, Alain Sarabia Pacis and Christopher E. Rudd
Figure S1: SC79 decreases anti-PD-1 resistant B16 tumor. (a) SC79 decreases B16 tumours. (b) SC79 decreases growth of EMT-6 syngeneic triple negative breast cancer tumor model.
Figure S2: Uncut western blots of anti-pAKT, anti-AKT and anti-actin for Figure 2. Fig. 2a: Left panel shows full sized blot with anti-pAKT, anti-AKT and anti-actin on which Figure 2a was based. The panels do not represent the full gel/blot but represents the majority of the gel (approximately 80%). NO material was visible in the remaining 20% which was cut off. Fig. 2b: Panels show full sized blot with anti-pAKT (left) and anti-AKT (right) on which Figure 2b was based. Left panel is underexposed showing a light background. The cross marking shows the approximate edge of blotting paper and gel transferred. Right panel shows more clearly the boundaries of the gel and transfer to the blotting paper. Anti-AKT stains at 60Kd band that comigrates with that stained with anti-pAKT. Anti-AKT also cross-reacts with a lower Mr band whose identify is unknown.
Figure S3: Gating strategies in CyTOF analysis (Panel a) and in flow cytometry (Panel b).

## Slide 2
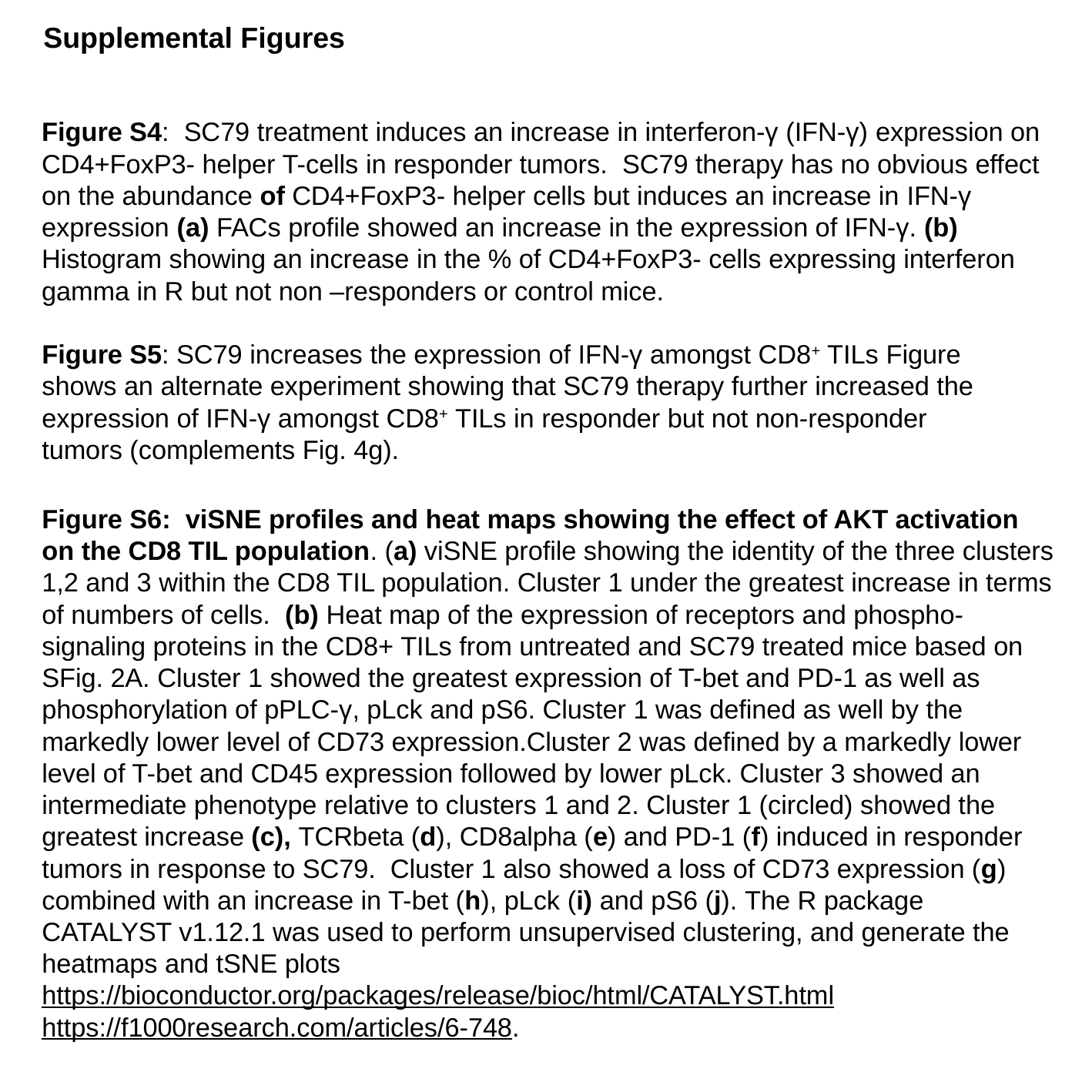

Supplemental Figures
Figure S4: SC79 treatment induces an increase in interferon-γ (IFN-γ) expression on CD4+FoxP3- helper T-cells in responder tumors. SC79 therapy has no obvious effect on the abundance of CD4+FoxP3- helper cells but induces an increase in IFN-γ expression (a) FACs profile showed an increase in the expression of IFN-γ. (b) Histogram showing an increase in the % of CD4+FoxP3- cells expressing interferon gamma in R but not non –responders or control mice.
Figure S5: SC79 increases the expression of IFN-γ amongst CD8+ TILs Figure shows an alternate experiment showing that SC79 therapy further increased the expression of IFN-γ amongst CD8+ TILs in responder but not non-responder tumors (complements Fig. 4g).
Figure S6: viSNE profiles and heat maps showing the effect of AKT activation on the CD8 TIL population. (a) viSNE profile showing the identity of the three clusters 1,2 and 3 within the CD8 TIL population. Cluster 1 under the greatest increase in terms of numbers of cells. (b) Heat map of the expression of receptors and phospho-signaling proteins in the CD8+ TILs from untreated and SC79 treated mice based on SFig. 2A. Cluster 1 showed the greatest expression of T-bet and PD-1 as well as phosphorylation of pPLC-γ, pLck and pS6. Cluster 1 was defined as well by the markedly lower level of CD73 expression.Cluster 2 was defined by a markedly lower level of T-bet and CD45 expression followed by lower pLck. Cluster 3 showed an intermediate phenotype relative to clusters 1 and 2. Cluster 1 (circled) showed the greatest increase (c), TCRbeta (d), CD8alpha (e) and PD-1 (f) induced in responder tumors in response to SC79. Cluster 1 also showed a loss of CD73 expression (g) combined with an increase in T-bet (h), pLck (i) and pS6 (j). The R package CATALYST v1.12.1 was used to perform unsupervised clustering, and generate the heatmaps and tSNE plots
https://bioconductor.org/packages/release/bioc/html/CATALYST.html
https://f1000research.com/articles/6-748.

## Slide 3
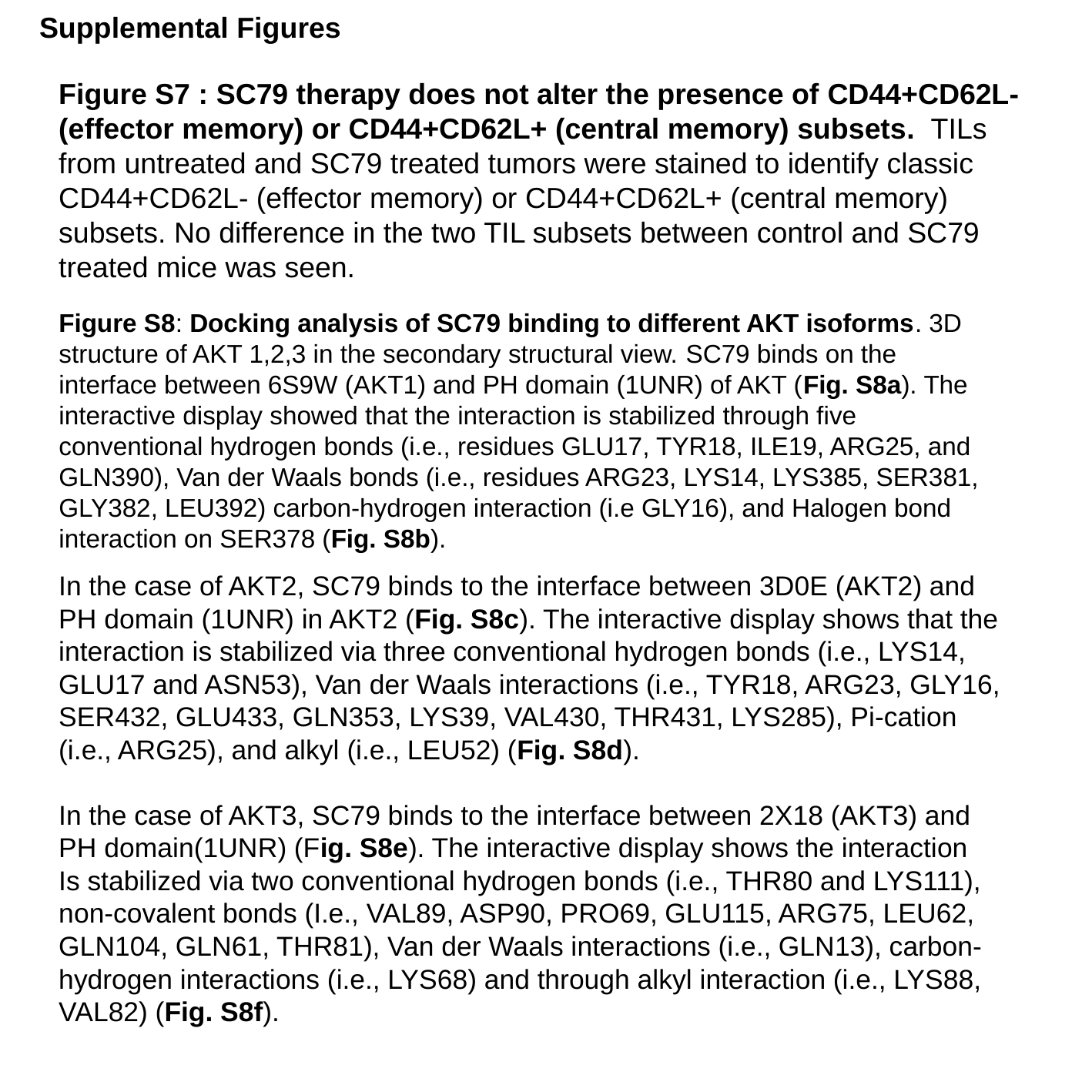

Supplemental Figures
Figure S7 : SC79 therapy does not alter the presence of CD44+CD62L- (effector memory) or CD44+CD62L+ (central memory) subsets. TILs from untreated and SC79 treated tumors were stained to identify classic CD44+CD62L- (effector memory) or CD44+CD62L+ (central memory) subsets. No difference in the two TIL subsets between control and SC79 treated mice was seen.
Figure S8: Docking analysis of SC79 binding to different AKT isoforms. 3D structure of AKT 1,2,3 in the secondary structural view. SC79 binds on the interface between 6S9W (AKT1) and PH domain (1UNR) of AKT (Fig. S8a). The interactive display showed that the interaction is stabilized through five conventional hydrogen bonds (i.e., residues GLU17, TYR18, ILE19, ARG25, and GLN390), Van der Waals bonds (i.e., residues ARG23, LYS14, LYS385, SER381, GLY382, LEU392) carbon-hydrogen interaction (i.e GLY16), and Halogen bond interaction on SER378 (Fig. S8b).
In the case of AKT2, SC79 binds to the interface between 3D0E (AKT2) and PH domain (1UNR) in AKT2 (Fig. S8c). The interactive display shows that the interaction is stabilized via three conventional hydrogen bonds (i.e., LYS14, GLU17 and ASN53), Van der Waals interactions (i.e., TYR18, ARG23, GLY16, SER432, GLU433, GLN353, LYS39, VAL430, THR431, LYS285), Pi-cation (i.e., ARG25), and alkyl (i.e., LEU52) (Fig. S8d).
In the case of AKT3, SC79 binds to the interface between 2X18 (AKT3) and PH domain(1UNR) (Fig. S8e). The interactive display shows the interaction
Is stabilized via two conventional hydrogen bonds (i.e., THR80 and LYS111), non-covalent bonds (I.e., VAL89, ASP90, PRO69, GLU115, ARG75, LEU62, GLN104, GLN61, THR81), Van der Waals interactions (i.e., GLN13), carbon-hydrogen interactions (i.e., LYS68) and through alkyl interaction (i.e., LYS88, VAL82) (Fig. S8f).

## Slide 4
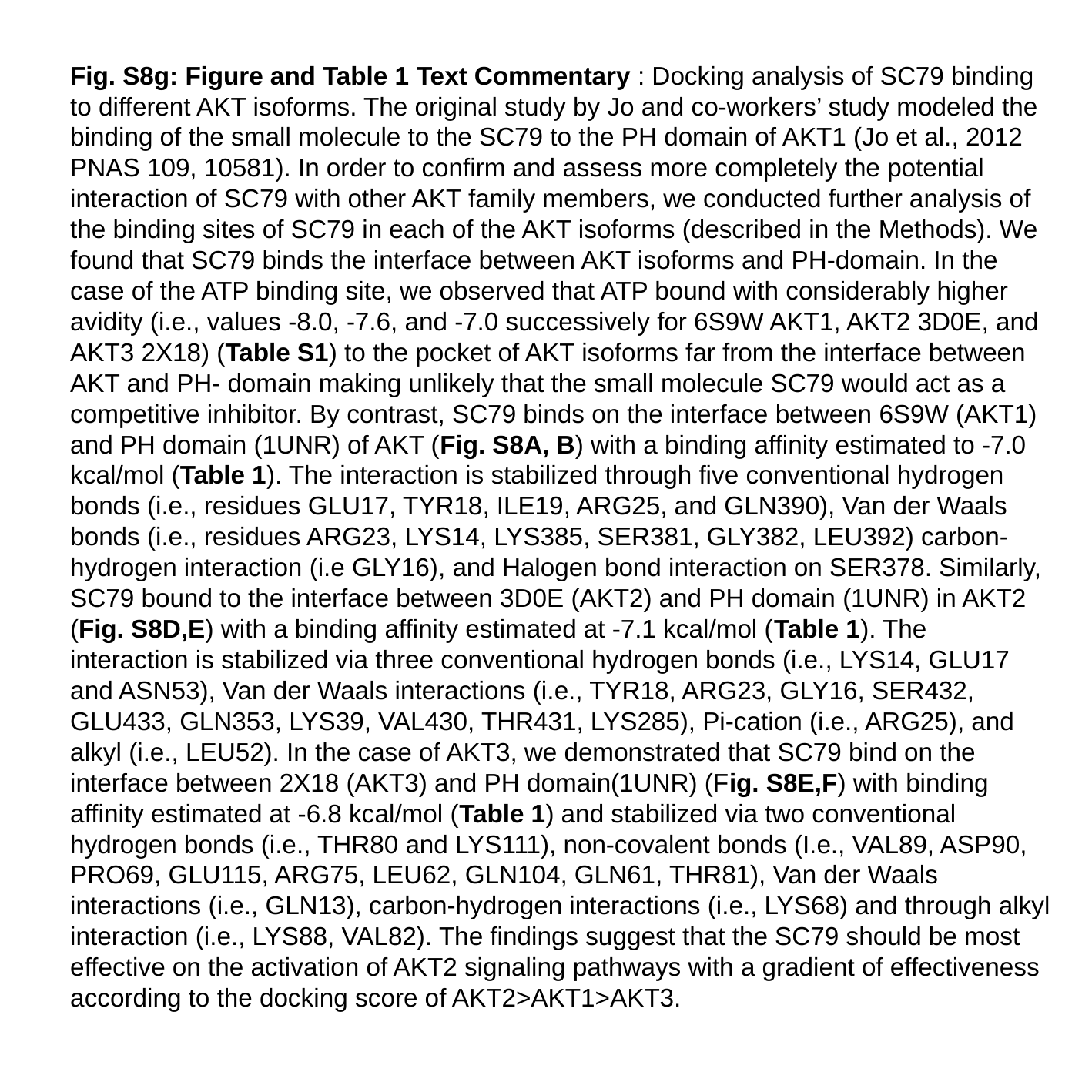

Fig. S8g: Figure and Table 1 Text Commentary : Docking analysis of SC79 binding to different AKT isoforms. The original study by Jo and co-workers’ study modeled the binding of the small molecule to the SC79 to the PH domain of AKT1 (Jo et al., 2012 PNAS 109, 10581). In order to confirm and assess more completely the potential interaction of SC79 with other AKT family members, we conducted further analysis of the binding sites of SC79 in each of the AKT isoforms (described in the Methods). We found that SC79 binds the interface between AKT isoforms and PH-domain. In the case of the ATP binding site, we observed that ATP bound with considerably higher avidity (i.e., values -8.0, -7.6, and -7.0 successively for 6S9W AKT1, AKT2 3D0E, and AKT3 2X18) (Table S1) to the pocket of AKT isoforms far from the interface between AKT and PH- domain making unlikely that the small molecule SC79 would act as a competitive inhibitor. By contrast, SC79 binds on the interface between 6S9W (AKT1) and PH domain (1UNR) of AKT (Fig. S8A, B) with a binding affinity estimated to -7.0 kcal/mol (Table 1). The interaction is stabilized through five conventional hydrogen bonds (i.e., residues GLU17, TYR18, ILE19, ARG25, and GLN390), Van der Waals bonds (i.e., residues ARG23, LYS14, LYS385, SER381, GLY382, LEU392) carbon-hydrogen interaction (i.e GLY16), and Halogen bond interaction on SER378. Similarly, SC79 bound to the interface between 3D0E (AKT2) and PH domain (1UNR) in AKT2 (Fig. S8D,E) with a binding affinity estimated at -7.1 kcal/mol (Table 1). The interaction is stabilized via three conventional hydrogen bonds (i.e., LYS14, GLU17 and ASN53), Van der Waals interactions (i.e., TYR18, ARG23, GLY16, SER432, GLU433, GLN353, LYS39, VAL430, THR431, LYS285), Pi-cation (i.e., ARG25), and alkyl (i.e., LEU52). In the case of AKT3, we demonstrated that SC79 bind on the interface between 2X18 (AKT3) and PH domain(1UNR) (Fig. S8E,F) with binding affinity estimated at -6.8 kcal/mol (Table 1) and stabilized via two conventional hydrogen bonds (i.e., THR80 and LYS111), non-covalent bonds (I.e., VAL89, ASP90, PRO69, GLU115, ARG75, LEU62, GLN104, GLN61, THR81), Van der Waals interactions (i.e., GLN13), carbon-hydrogen interactions (i.e., LYS68) and through alkyl interaction (i.e., LYS88, VAL82). The findings suggest that the SC79 should be most effective on the activation of AKT2 signaling pathways with a gradient of effectiveness according to the docking score of AKT2>AKT1>AKT3.

## Slide 5
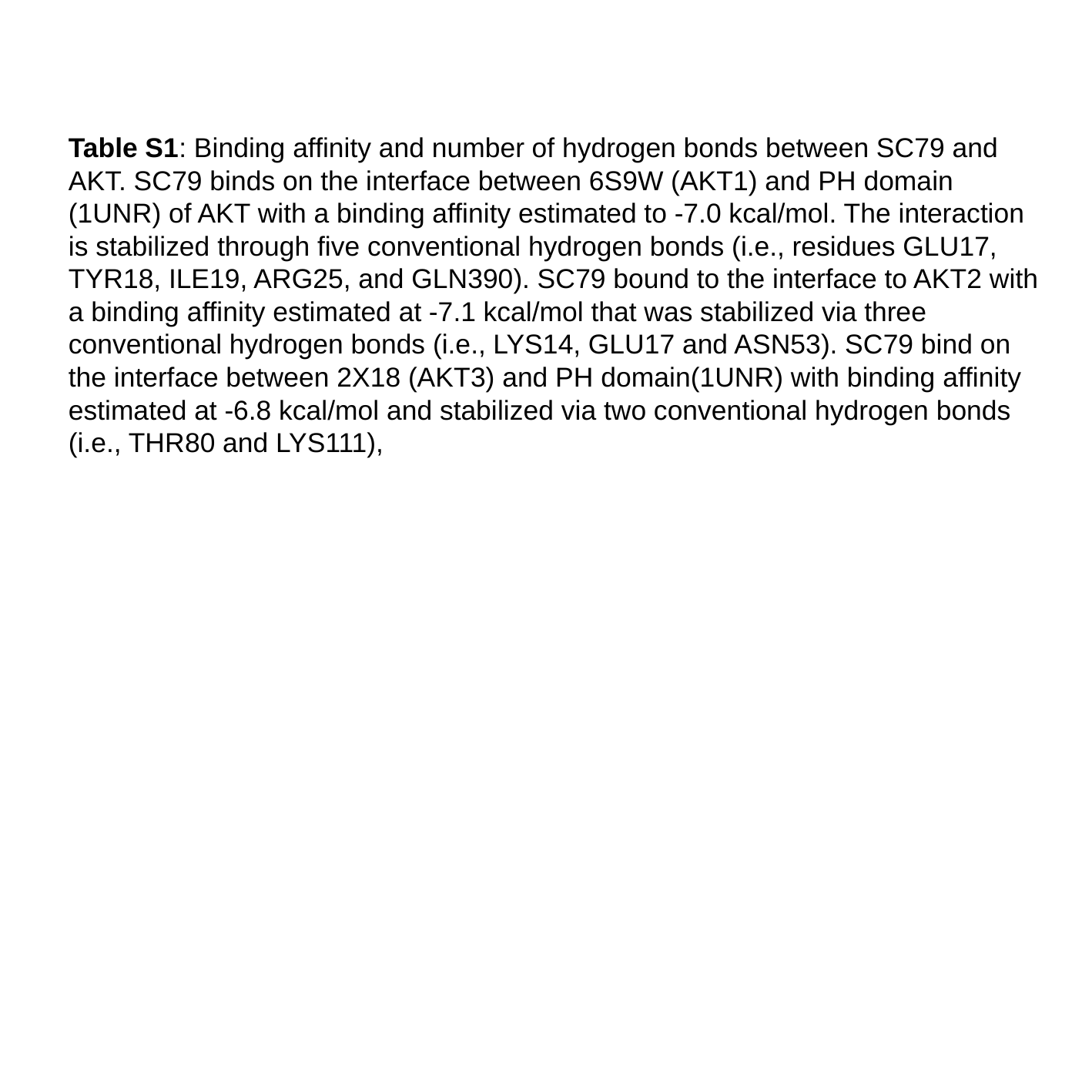

Table S1: Binding affinity and number of hydrogen bonds between SC79 and AKT. SC79 binds on the interface between 6S9W (AKT1) and PH domain (1UNR) of AKT with a binding affinity estimated to -7.0 kcal/mol. The interaction is stabilized through five conventional hydrogen bonds (i.e., residues GLU17, TYR18, ILE19, ARG25, and GLN390). SC79 bound to the interface to AKT2 with a binding affinity estimated at -7.1 kcal/mol that was stabilized via three conventional hydrogen bonds (i.e., LYS14, GLU17 and ASN53). SC79 bind on the interface between 2X18 (AKT3) and PH domain(1UNR) with binding affinity estimated at -6.8 kcal/mol and stabilized via two conventional hydrogen bonds (i.e., THR80 and LYS111),

## Slide 6
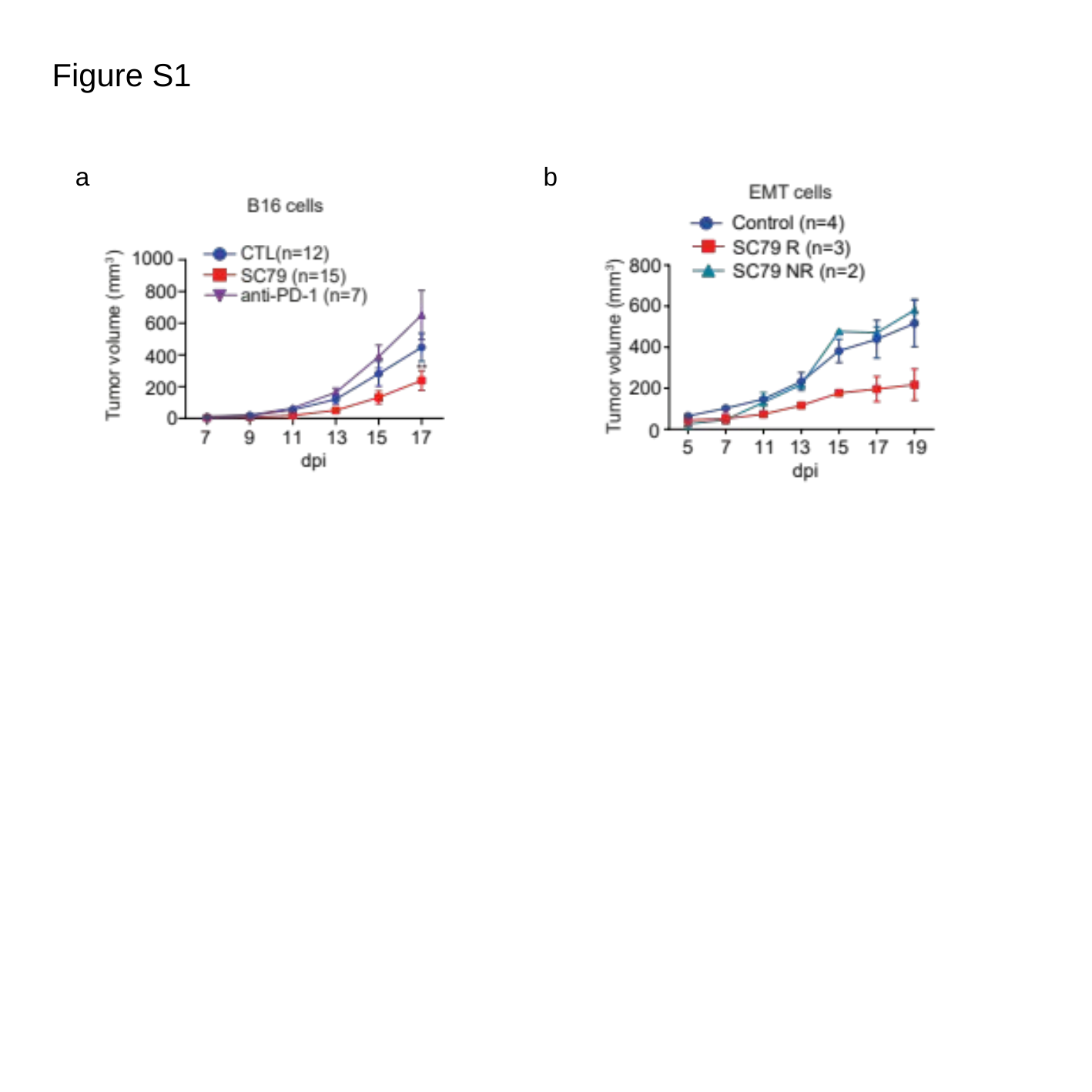

Figure S1
a
b

## Slide 7
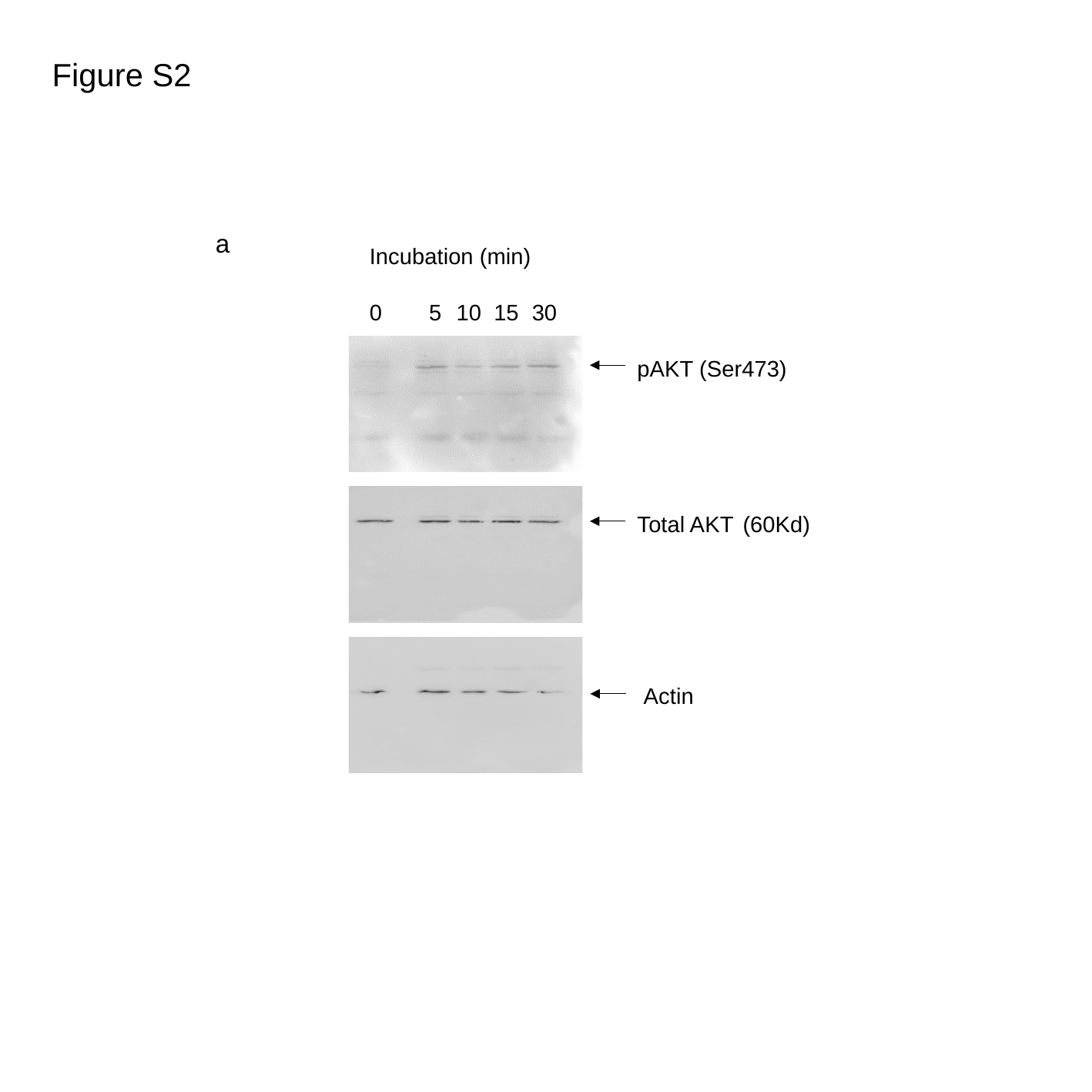

Figure S2
a
Incubation (min)
0
5
10
15
30
pAKT (Ser473)
(60Kd)
Total AKT
Actin

## Slide 8
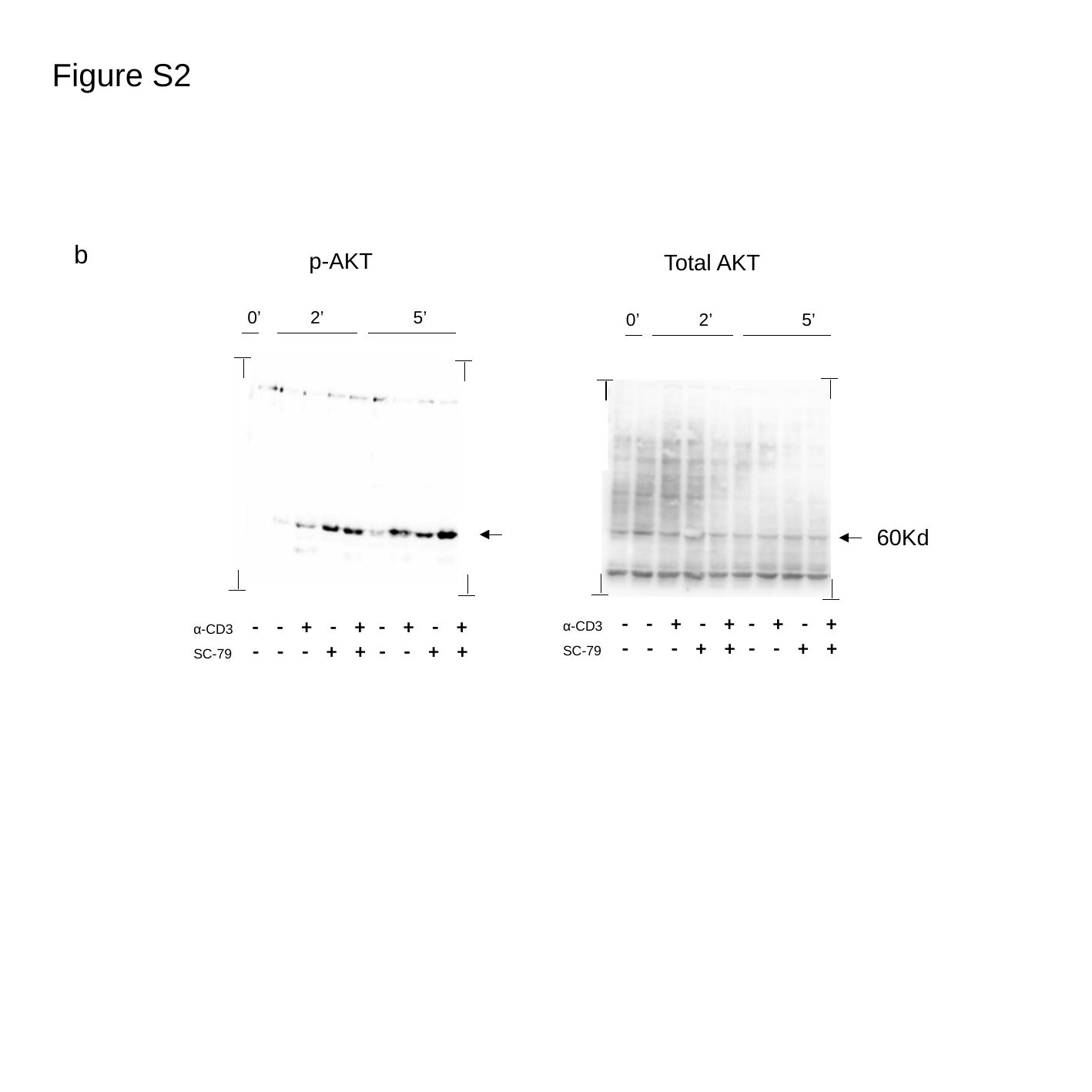

Figure S2
b
p-AKT
Total AKT
 0’ 2’ 5’
 0’ 2’ 5’
60Kd
α-CD3 - - + - + - + - +
SC-79 - - - + + - - + +
α-CD3 - - + - + - + - +
SC-79 - - - + + - - + +

## Slide 9
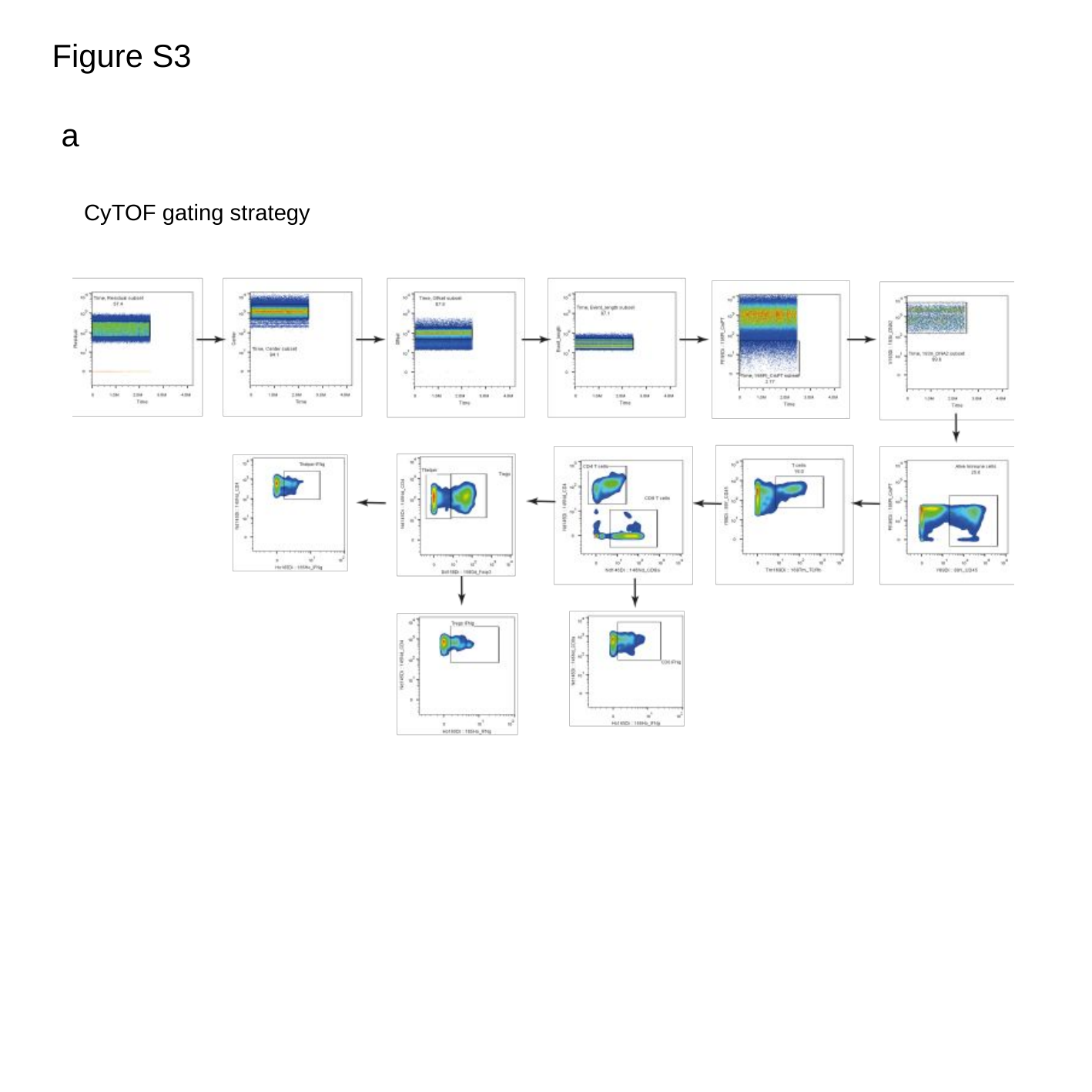

Figure S3
a
CyTOF gating strategy

## Slide 10
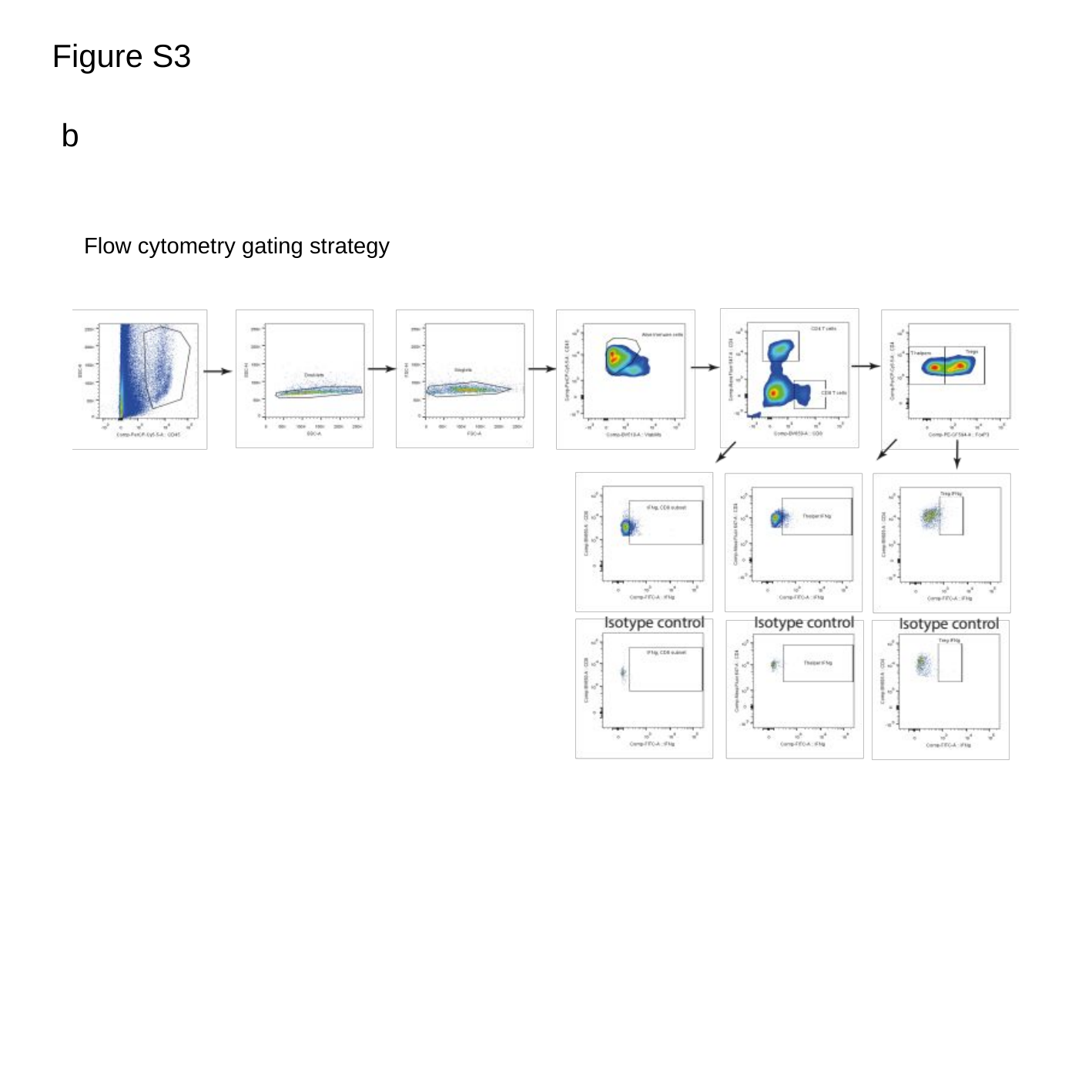

Figure S3
b
Flow cytometry gating strategy

## Slide 11
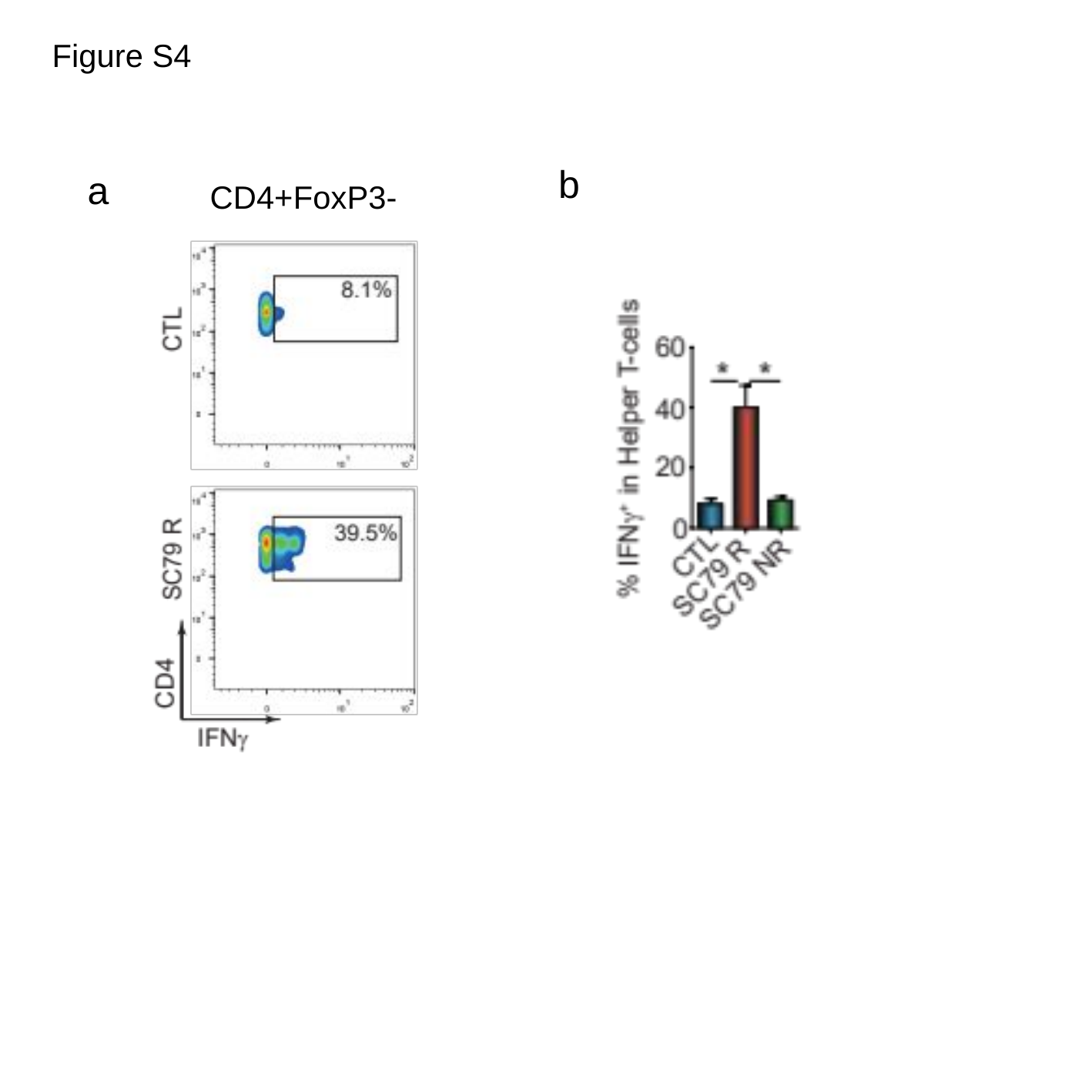

Figure S4
b
a
CD4+FoxP3-

## Slide 12
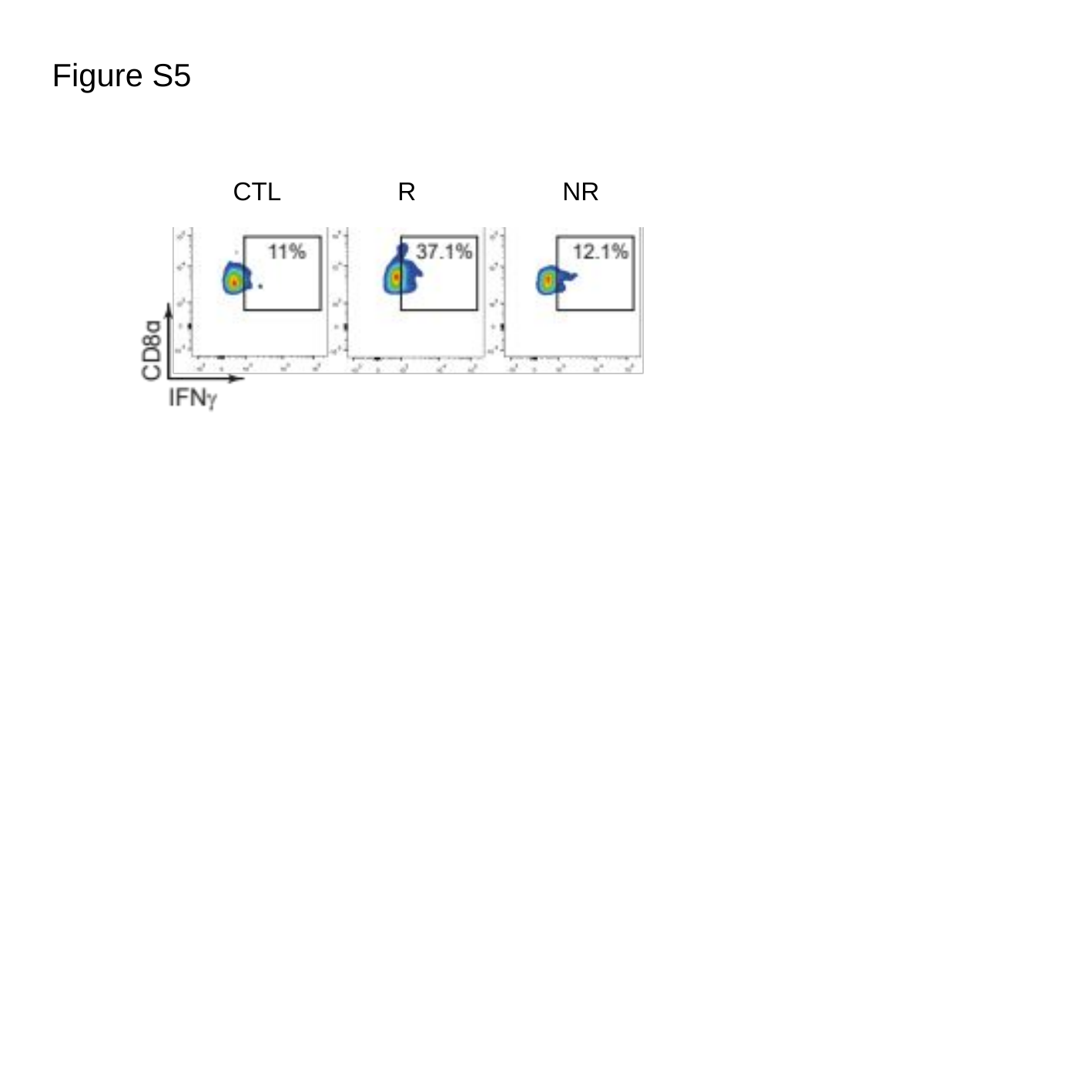

Figure S5
CTL
R
NR

## Slide 13
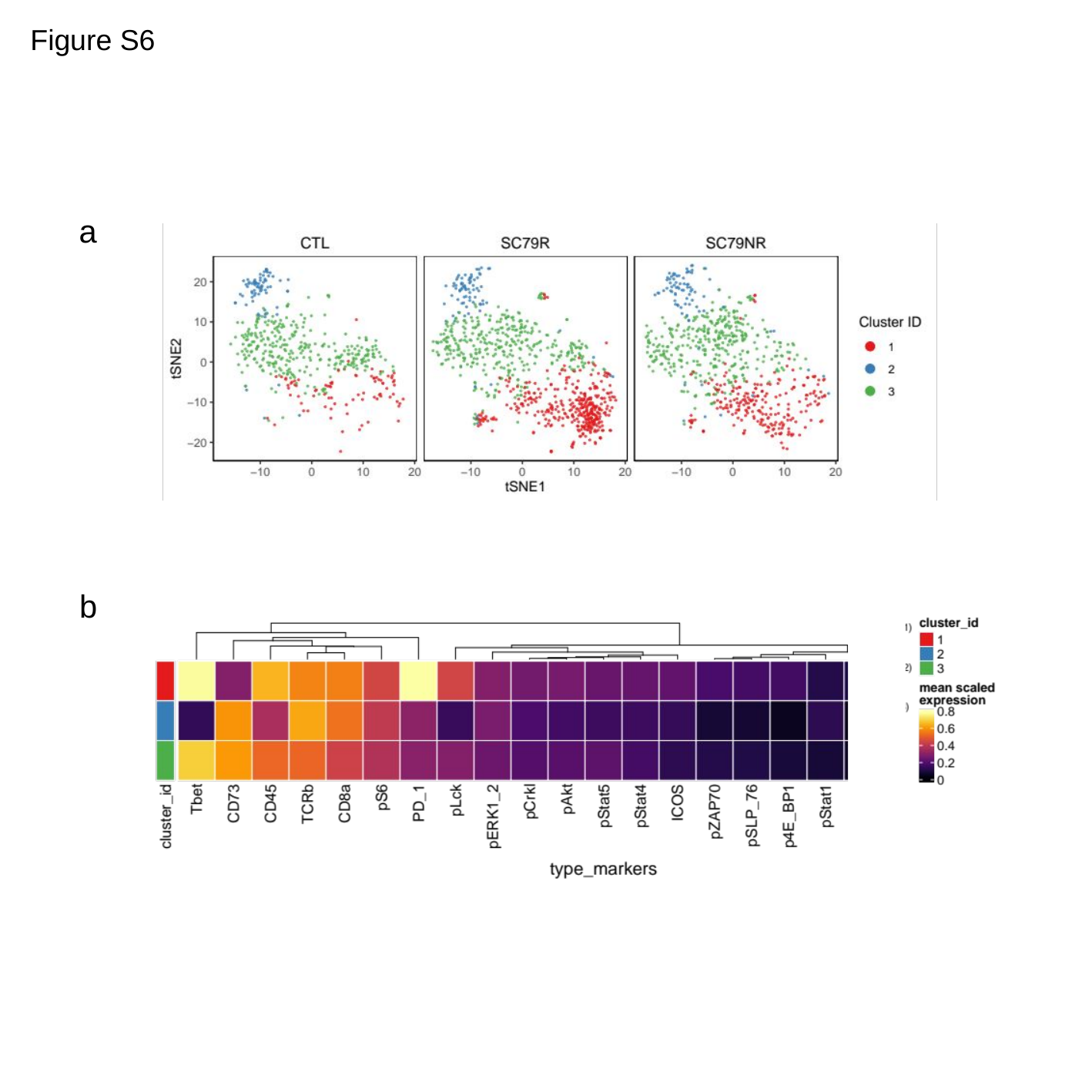

Figure S6
a
b

## Slide 14
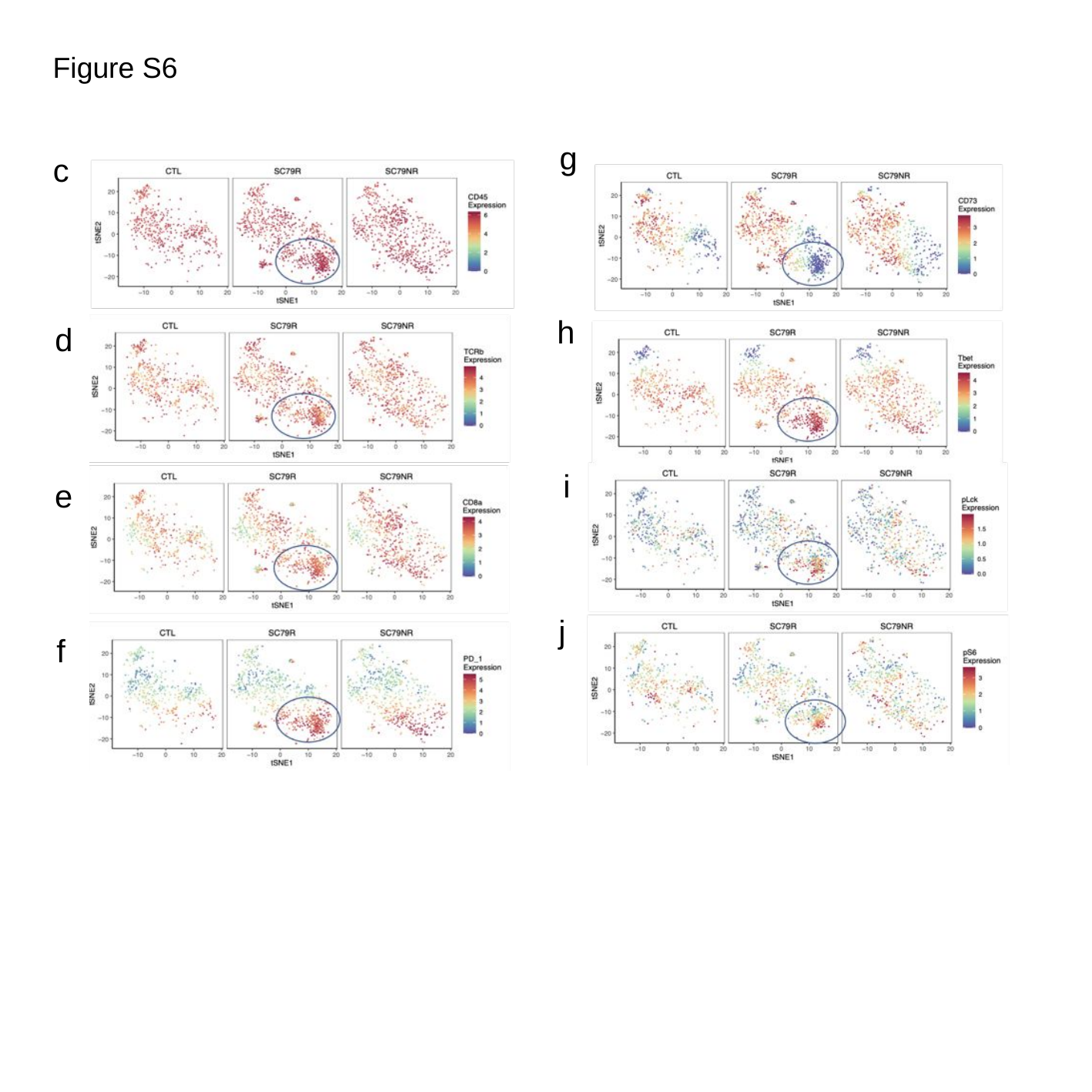

Figure S6
g
c
h
d
i
e
j
f

## Slide 15
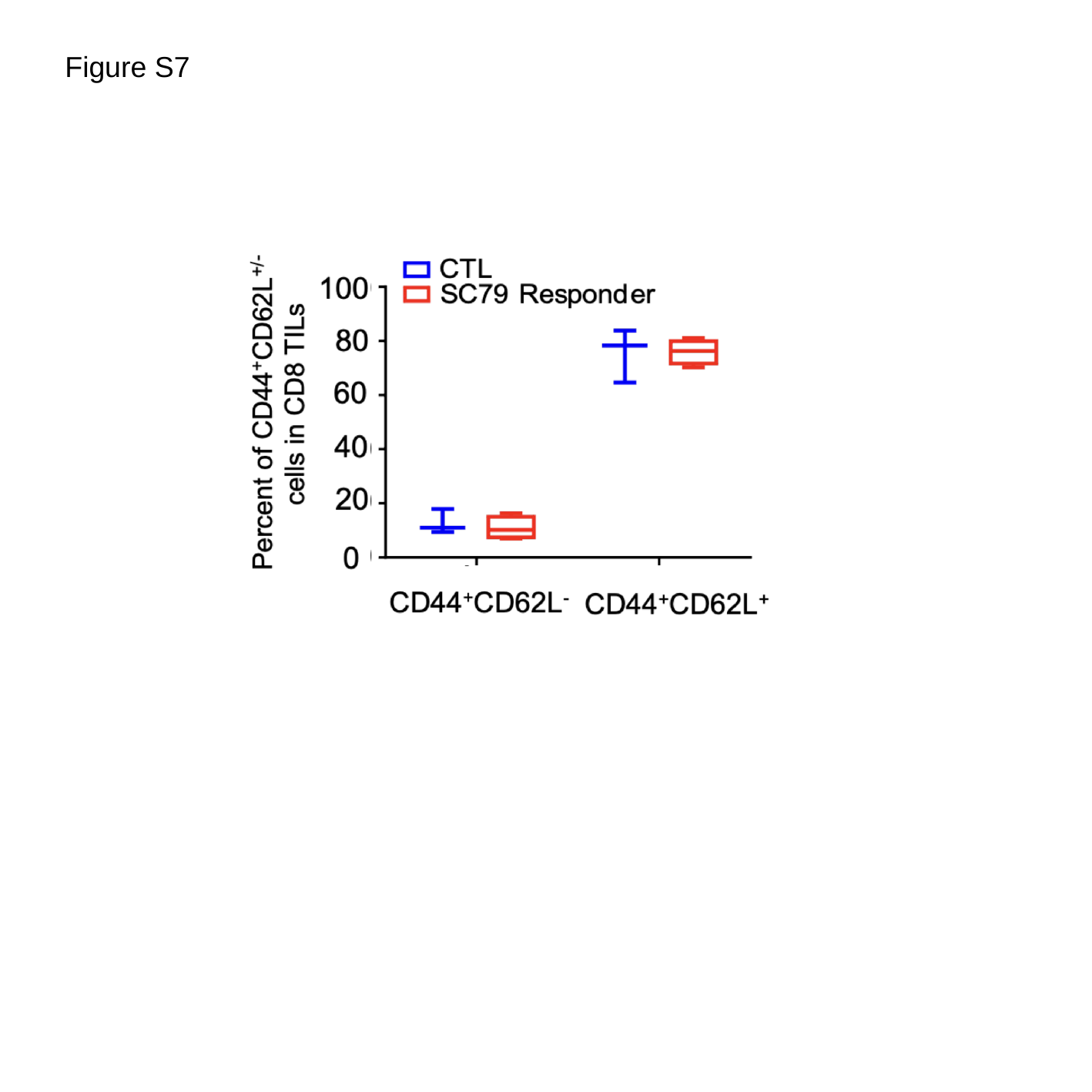

Figure S7

## Slide 16
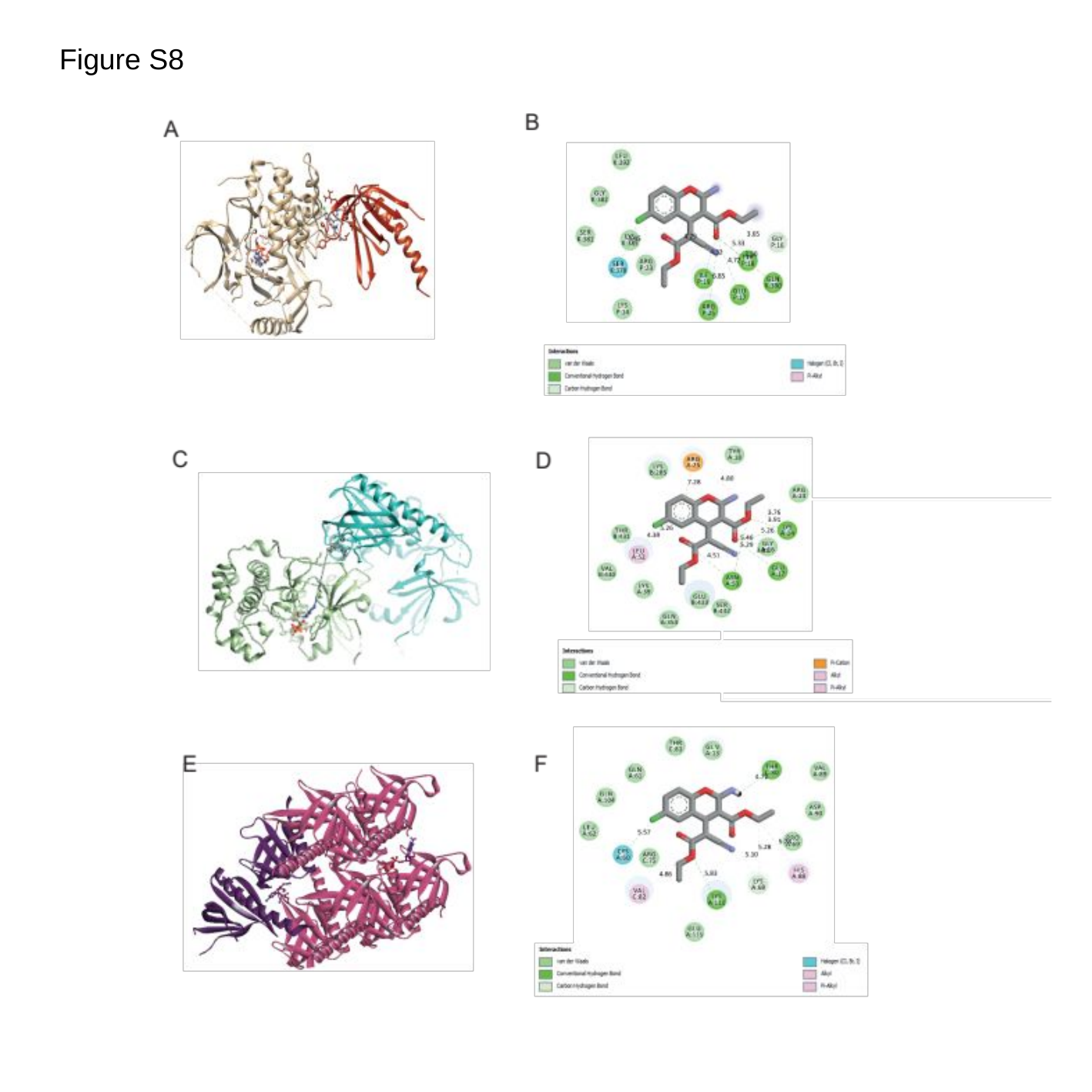

Figure S8

## Slide 17
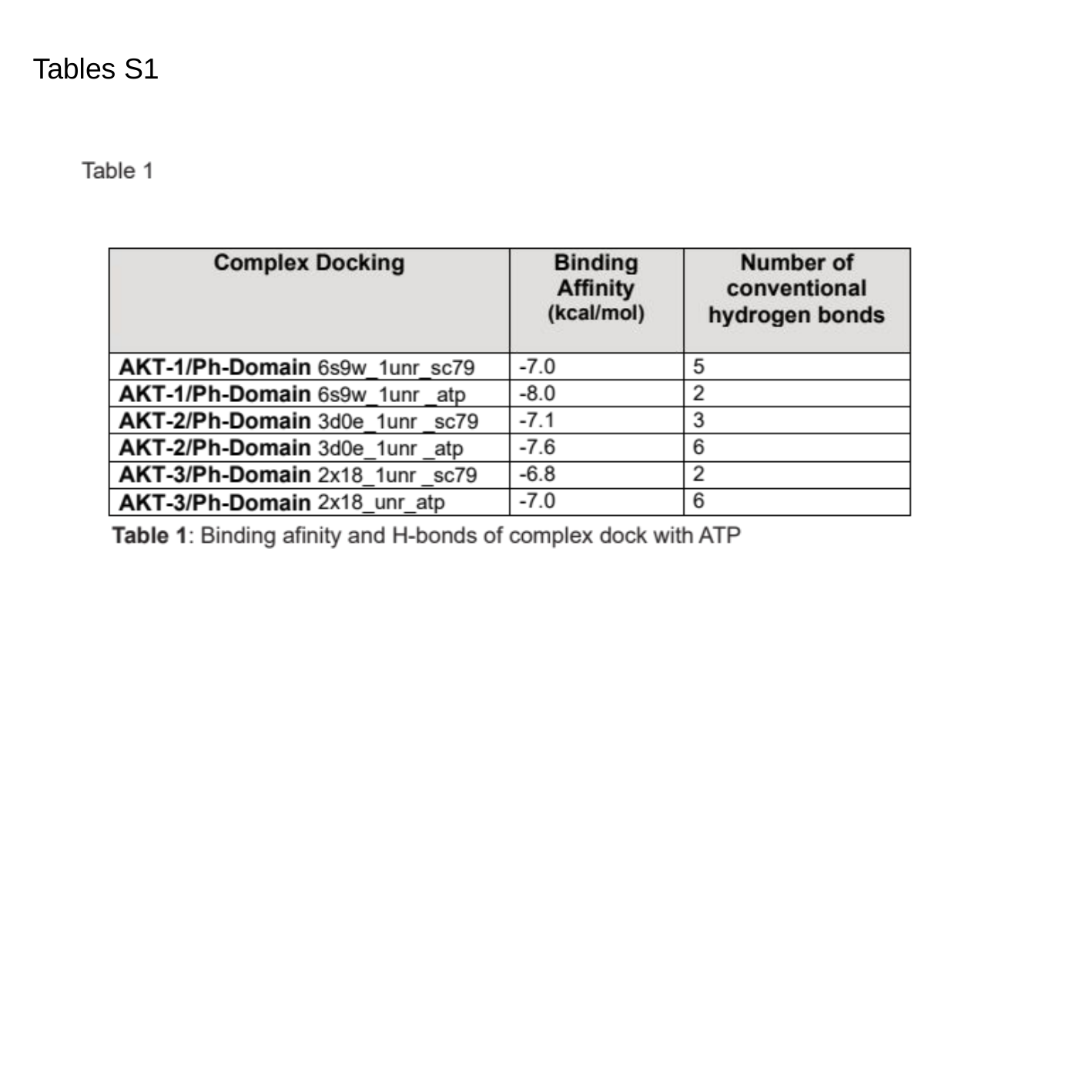

Tables S1
